# Parkinson disease related ATP13A2 evolved early in animal evolution

**DOI:** 10.1371/journal.pone.0193228

**Published:** 2018-03-05

**Authors:** Danny Mollerup Sørensen, Tine Holemans, Sarah van Veen, Shaun Martin, Tugce Arslan, Ida Winther Haagendahl, Henrik Waldal Holen, Norin Nabil Hamouda, Jan Eggermont, Michael Palmgren, Peter Vangheluwe

**Affiliations:** 1 Laboratory of Cellular Transport Systems, Department of Cellular and Molecular Medicine, KU Leuven; Leuven, Belgium; 2 Department of Plant and Environmental Sciences, University of Copenhagen, Frederiksberg C, Denmark; Van Andel Institute, UNITED STATES

## Abstract

Several human P5-type transport ATPases are implicated in neurological disorders, but little is known about their physiological function and properties. Here, we investigated the relationship between the five mammalian P5 isoforms ATP13A1-5 in a comparative study. We demonstrated that ATP13A1-4 isoforms undergo autophosphorylation, which is a hallmark P-type ATPase property that is required for substrate transport. A phylogenetic analysis of P5 sequences revealed that ATP13A1 represents clade P5A, which is highly conserved between fungi and animals with one member in each investigated species. The ATP13A2-5 isoforms belong to clade P5B and diversified from one isoform in fungi and primitive animals to a maximum of four in mammals by successive gene duplication events in vertebrate evolution. We revealed that ATP13A1 localizes in the endoplasmic reticulum (ER) and experimentally demonstrate that ATP13A1 likely contains 12 transmembrane helices. Conversely, ATP13A2-5 isoforms reside in overlapping compartments of the endosomal system and likely contain 10 transmembrane helices, similar to what was demonstrated earlier for ATP13A2. *ATP13A1* complemented a deletion of the yeast P5A ATPase *SPF1*, while none of *ATP13A2-5* could complement either the loss of *SPF1* or that of the single P5B ATPase *YPK9* in yeast. Thus, ATP13A1 carries out a basic ER function similar to its yeast counterpart Spf1p that plays a role in ER related processes like protein folding and processing. ATP13A2-5 isoforms diversified in mammals and are expressed in the endosomal system where they may have evolved novel complementary or partially redundant functions. While most P5-type ATPases are widely expressed, some P5B-type ATPases (ATP13A4 and ATP13A5) display a more limited tissue distribution in the brain and epithelial glandular cells, where they may exert specialized functions. At least some P5B isoforms are of vital importance for the nervous system, since ATP13A2 and ATP13A4 are linked to respectively Parkinson disease and autism spectrum disorders.

## Introduction

P-type ATPases are intrinsic membrane proteins that couple the hydrolysis of ATP to the active transport of substrates over biological membranes [1]. The large family of P-type ATPases comprises five subfamilies (P1-P5) with a total of 36 human isoforms [2]. Phylogenetic analysis of P-type ATPase sequences in early genomic sequences revealed the existence of a fifth P-type ATPase subfamily, which is exclusively found in eukaryotic lineages [2]. However, the substrate specificity and exact cellular role of the P5-type ATPases remain elusive [1, 3, 4]. So far, various substrates for the P5 ATPases were proposed, ranging from ion transport (*e*.*g*. Ca^2+^, Mg^2+^, Mn^2+^ or Zn^2+^) to polyamines, sterols or other lipids, but conclusive evidence has not yet been presented [5–18].

Despite their elusive molecular function, two of the five human P5 ATPases, *i*.*e*. ATP13A2 and ATP13A4, have attracted considerable interest as they are implicated in neurological disorders. Loss-of-function mutations in ATP13A2 (PARK9, OMIM 610513) are associated with the etiology of Kufor-Rakeb syndrome (KRS), a severe juvenile onset autosomal recessive form of Parkinson Disease (PD) with dementia [19–25], hereditary spastic paraplegia [26] and the lysosomal storage disorder neuronal ceroid lipofuscinosis [27–30]. In various model organisms and cell systems, ATP13A2 renders protection against well-known genetic and environmental risk factors of PD, such as α-synuclein toxicity [5] and Fe^3+^- [31], Zn^2+^- [6, 7, 11] or Mn^2+^-toxicity [10, 13, 14, 32, 33], respectively. In addition, ATP13A2 protects against mitochondrial stress [7, 14, 15, 34, 35], *e*.*g*. induced by complex I inhibition [4, 36, 37].

In one patient with expressive and receptive language delay, the *ATP13A4* gene was disrupted by an inversion on chromosome 3q [38]. In addition, several individuals with autism spectrum disorders carried a mutation in the coding region of the *ATP13A4* gene, substituting D646 for E646 [39]. Two other studies point to a putative link between ATP13A4 and epileptic encephalopathies [40] or childhood apraxia of speech [41].

The expression pattern of the P5-type ATPases was previously studied in mice. The detection of Atp13a1 and Atp13a2 mRNAs in all examined mouse tissue points to a housekeeping function of both isoforms [42], although tissue differences in expression levels may indicate that they may serve organ-specific functions as well. A particularly high Atp13a2 expression was found in the brain, whereas Atp13a1 levels were low in the heart. Atp13a3 also showed a widespread expression, with the highest levels in liver, while Atp13a4 and Atp13a5 expression was more restricted to brain and stomach [42]. Atp13a1-4 transcripts were differentially expressed in the cerebral cortex, hippocampus, brainstem and cerebellum and during mouse development, *Atp13a1* and *Atp13a2* transcript expression was highest at the peak of neurogenesis (E15) [43]. These studies, together with their emerging role in neurological disorders point to an important function of P5 ATPases in the brain.

Despite their relevance, most of the mammalian P5 isoforms remain largely unexplored and information on their relationship, molecular properties and subcellular localization is scarce or non-existing. Therefore, we performed a comparative study to investigate the phylogenetic relation, subcellular localization and functional properties of the five mammalian P5-ATPase isoforms. Based on these results, we describe their evolutionary paths and formulate the possible physiological context of this elusive group of primary pumps in animals.

## Materials and methods

### Phylogenetic analysis

Protein sequences with significant similarity (Expect value <e^-30^) to the *Homo sapiens* P5A ATPase ATP13A1 were first identified using the Basic Local Alignment Search Tool (BLAST) for sequences in completed genomes in the NCBI non-redundant protein sequence database (http://blast.ncbi.nlm.nih.gov/) and in EchinoBase (http://www.echinobase.org/Echinobase/). P5 ATPases in this dataset were first identified by the presence of fully intact signature motifs characteristic for P-type ATPases [2] and for P5-type ATPases specifically [44]. This was further confirmed by construction of separate phylogenetic trees for all candidate sequences in each individual genome together with a set of known P-type ATPases including *H*. *sapiens* ATP13A1-5 using first MUSCLE alignment [45] and then by maximum likelihood phylogeny reconstruction in a Gamma distributed LG model (see below), using MEGA6 software. As most genomes are still draft versions in the databases, this approach allowed for identification and removal of duplicates and sequences that do not represent complete proteins. The final dataset was extended by collecting annotated P5-ATPase sequences from the KEGG database (http://www.genome.jp/tools-bin/search_sequence?prog=blast&db=cmy&seqid=aa). The resulting dataset comprised 146 sequences from 43 genomes, which represented different stages in the evolution of animals. Accession numbers are listed in S1 Table.

**Fig 1:**
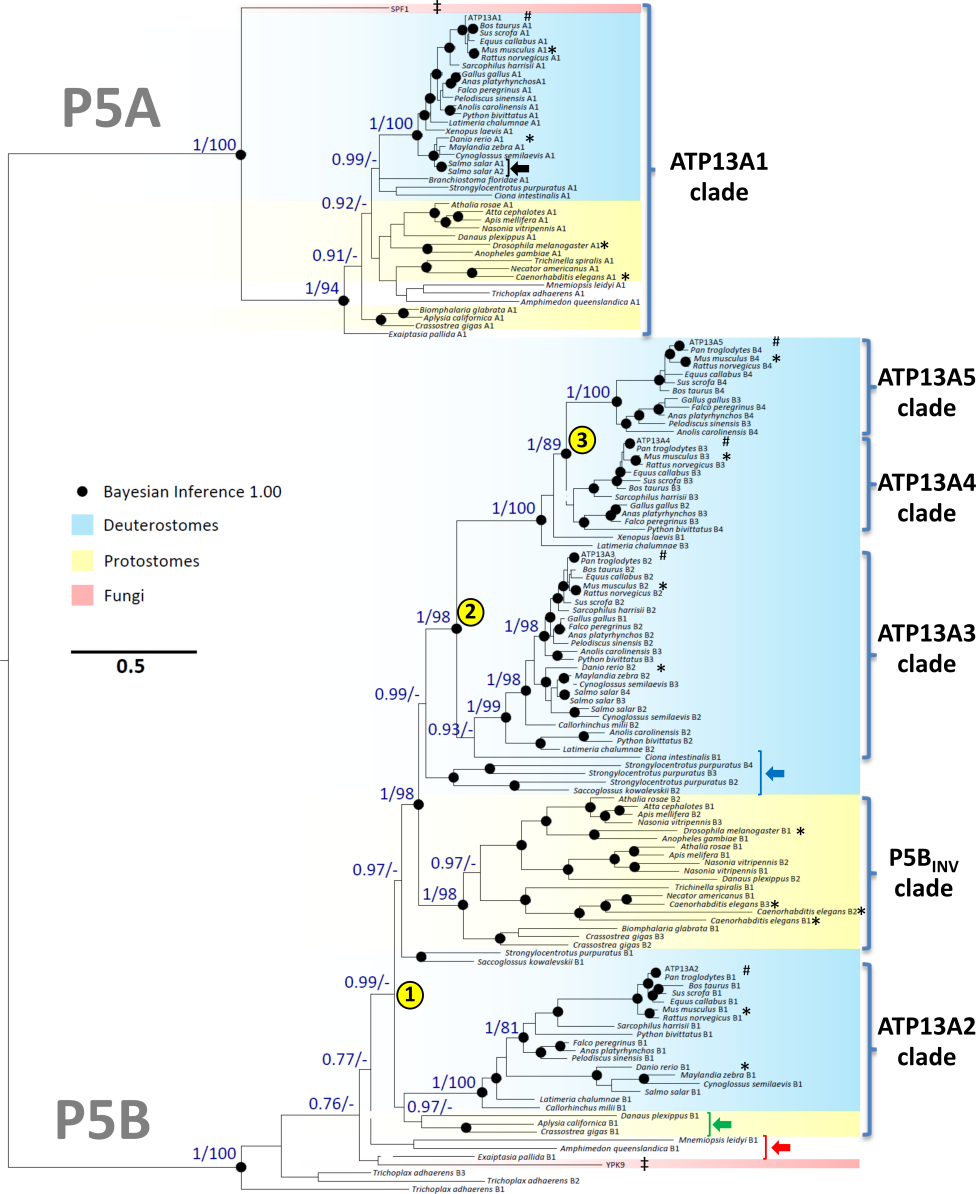
Phylogenetic analysis of the P5-type ATPases. Phylogenetic tree of 146 protein sequences of P5-type ATPases as inferred from the combined output of two independent statistical methods of measurement (Bayesian inference and maximum likelihood analysis, see materials and methods for a detailed description). Yellow and blue shades represent sequences from protostomia and deuterostomia, respectively. Black dots represent Bayesian inference values of 1.00 and numbers noted at key nodes are represented with Bayesian inference statistical values on the *left* and maximum likelihood statistical values on the *right* of the dash (*left*/*right*). Accession numbers of named sequences can be found in S1 Table. Positions of human and model animal organisms (*C*. *elegans*, *D*. *melanogaster*, *D*. *rerio*, *M*. *musculus*) are marked by # and *, respectively. Yeast sequences are marked by ‡. The meaning of the colored arrows is provided in the description of the main text. The yellow numbers indicate the three major P5B gene duplications in vertebrate evolution as discussed in the main text.

**Fig 2:**
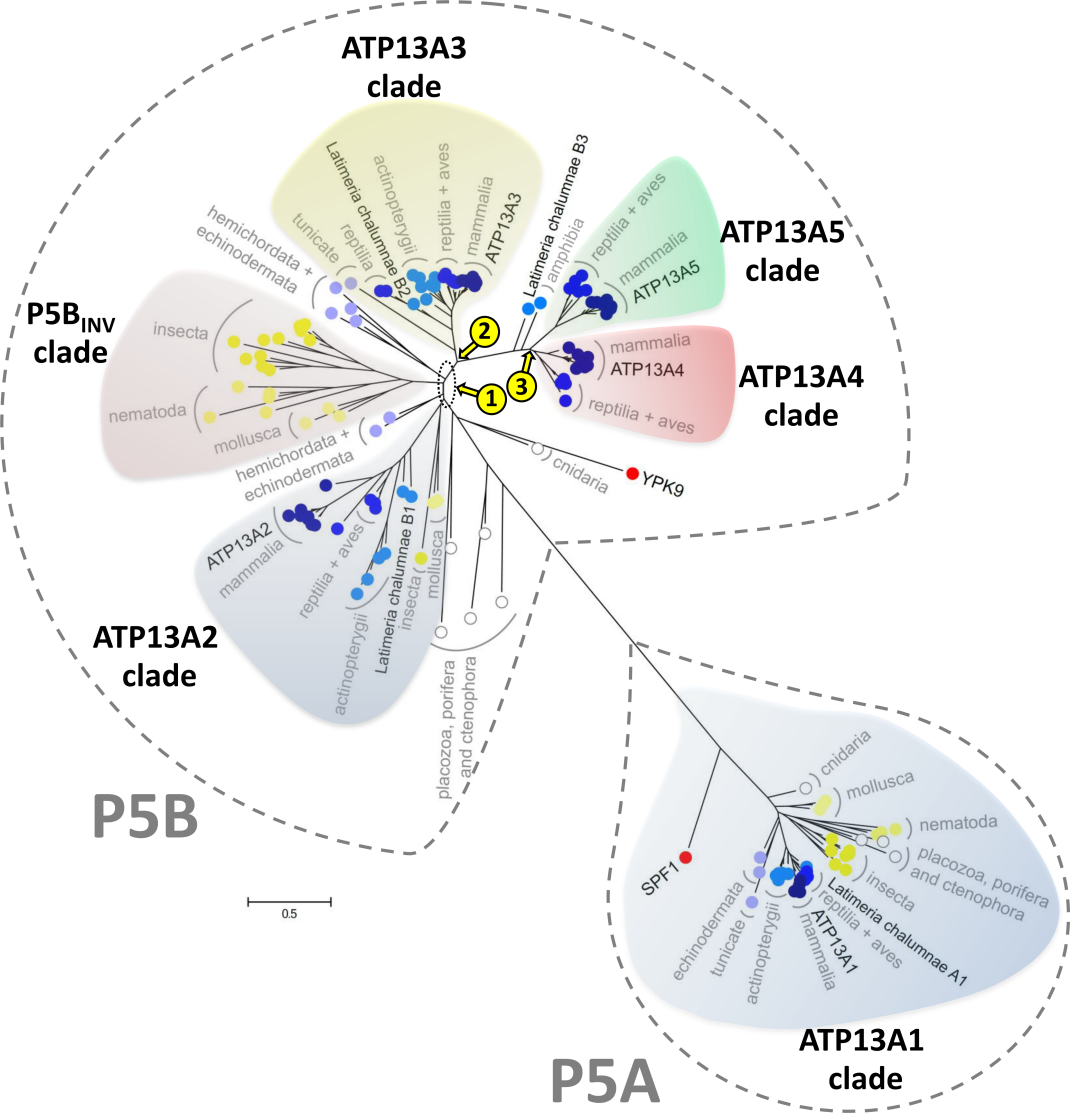
P5-type ATPase phylogenetic tree. Simplified view of the phylogenetic tree calculated in Fig 1. The P5A group includes only ATP13A1-like sequences (grey), while the P5B group includes subclades belonging to the ATP13A2 (blue), P5B_inv_ (purple, invertebrate P5B), ATP13A3 (yellow), ATP13A4 (red) and ATP13A5 (green) clades. Sequences from cnidaria, placozoa and ctenophore are marked in white dots. Invertebrate sequences are marked in yellow dots. Sequences from hemichordates and echinoderms are marked with light blue dots and sequences from higher vertebrates are marked in progressively darker shaded blue dots. Yeast Spf1p and Ypk9p are marked with red dots. The yellow numbers indicate the three major P5B gene duplications in vertebrate evolution as discussed in the main text.

Protein alignment of the full-length P5-type ATPases sequences was performed with MUSCLE [45]. All positions containing gaps or ambiguous data were eliminated, leaving out a total of 743 positions in the final data set. The evolutionary history was inferred assuming an LG [46] + INVGAMMA model, as identified by ProtTest [47]. Phylogenetic analyses were subsequently conducted using Bayesian inference and maximum likelihood methods. Bayesian inference was performed with MrBayes 3.2.6 [48] and maximum likelihood analyses with RAxML 8.2.9 [49]. MrBayes analysis was performed using the following settings: eight chains of Markov chain Monte Carlo iterations and heated parameter of 0.05 with trees sampled every 100 generations. The average standard deviation of split frequencies at termination of the analysis, after 2,360,000 generations, was 0.009821 for trees in Fig 1 and Fig 2. Branching was confirmed in a tree based on the same dataset, but including the human Na^+^/K^+^-ATPase (ATP1A1), which was run for 8,000,000 generations and resulted in an average standard deviation of split frequencies of 0.009982, after 7,210,000 generations. The unrooted consensus tree of the MrBayes analysis was selected for presentation. In the RAxML analysis, clade robustness was assessed with 100 rapid bootstrap inferences followed by thorough analysis of maximum likelihood. Bootstrap values were inferred from 1000 replicates to obtain statistical support for the placement of nodes [50].

### DNA constructs

The human hATP13A2-pDONR221 (hATP13A2 splice variant 2) and YPK9-pDONR221 constructs were a kind gift of Dr. Lindquist [5]. The yeast *SPF1* construct was generated by cloning the *SPF1* cDNA from genomic DNA isolated from the BY4741 wild type strain and inserted into the pJET1.2 vector (ThermoFisher). The cDNA was further transferred in an *Xho*I/*Xba*I flanked cassette using polymerase chain reaction (PCR) and ligated into the YEp351 vector in front of the *PMA1* constitutive promotor using *Xho*I/*Spe*I digestion/ligation. The human hATP13A3-pENTR223.1 construct was purchased from DNASU Plasmid Repository (Arizona, USA). cDNAs of Atp13a1, Atp13a4 and Atp13a5 were picked up from a mouse brain cDNA library by PCR and inserted in a Gateway compatible pDONR221 vector. Catalytic dead mutants of ATP13A1-5, Ypk9p or Spf1p, in which the Asp acceptor residue for the catalytic autophosphoryation was mutated into an Asn, were created via site-directed mutagenesis (Quickchange II XL Site-directed mutagenesis, Stratagene). N-terminal constructs of ATP13A1 (Mb-Ma, 197 amino acids or Mb, 95 amino acids) and ATP13A2 splice variant 2 (Ma-M1, 251 amino acids or Ma, 187 amino acids) were generated via PCR. Constructs were subsequently transferred via the Gateway system (LifeTechnologies) to mammalian (pcDNA3.1, pcDNA6.2) or yeast (pAG425GDP and YEp351, which were made gateway compatible using the supplier's instructions) expression vectors containing N- or C-terminal GFP- or mCherry-tags or no tag in the case of YEp351. To produce tagged Ypk9p proteins for purification (Ypk9p WT and Ypk9p D781N), the cDNA was cloned from the pDONR221 plasmids using PCR cloning and placed in front of a GAL10 promotor in a yeast episomal vector (pRS423). A FLAG/RGS10xhis purification tag was inserted at the 5’ end of the cDNA by incorporating its coding sequence into the primers. All constructs were verified by sequencing.

### Cell culture

COS-8 cells were cultured in Dulbecco’s Modified Eagle Medium (DMEM) containing 10% Fetal Calf Serum (FCS), 4 mM GlutaMAX^TM^-I supplement, 100 U Penicillin/ml, 100 μg Streptomycin/ml and 0,1 mM MEM non-essential amino acids (Invitrogen) at 37°C in an atmosphere of air with 10% CO_2_. HeLa cells (human cervix adenocarcinoma cell line) were cultured in DMEM containing 10% FCS, 4 mM GlutaMAX^TM^-I supplement, 100 U Penicillin/ml, 100 μg Streptomycin/ml (Invitrogen) at 37°C in an atmosphere of air with 5% CO_2_. MEF (mouse embryonic fibroblast) and Neuro2a (mouse neuroblastoma) cells were cultured in DMEM/F-12 containing 10% FCS, 4 mM GlutaMAX^TM^-I supplement, 100 U Penicillin/ml, 100 μg Streptomycin/ml. Transfections were performed using GeneJuice (Novagen) according to the manufacturer’s protocol. Briefly, cells were split in 15 cm ø Petri dishes (4.1 10^6^ cells/dish), chamber slides (20.000 cells/chamber) or 12-well plates (20.000 cells/well) and after 24 h, transfections were performed using a concentration of 3 μl GeneJuice per μg DNA. 48 h after transfection, cells were harvested, examined under the microscope or prepared for immunostaining.

### Generation of an ATP13A1 antibody

An ATP13A1 antibody (SY2459) was generated against the peptide ^543^RGVAGLRDGKEVTPVc, which was conjugated to keyhole limpet hemocyanin (KHL). The antibody was produced by Eurogentec with the Rabit DX Speedy– 28-day immunization protocol (4 injections, 3 bleeds) and affinity purified.

### Immunoblotting

15 cm ø Petri dishes of transiently transfected COS-8 cells were harvested 48 h post transfection. Subcellular fractions were obtained after differential centrifugation as previously described [37]: the nuclear fraction (1,000 g, 10 min, 4°C), the mitochondrial/lysosomal fraction (12,000 g, 20 min, 4°C) and the microsomal fraction (200,000 g, 35 min, 4°C). To prepare total membrane fractions, the nuclear fraction was removed (1,000 g, 10 min, 4°C) and the remaining lysate was centrifuged for 35 min at 200,000 g (4°C). Pellets were suspended in 0.25 M sucrose supplemented with protease inhibitors (SigmaFast, Sigma). Protein concentrations were determined via the Qubit fluorometric method (LifeTechnologies).

Immunoblotting was performed as previously described [37]. Membranes were probed with primary rabbit polyclonal antibodies directed against ATP13A1 (SY2559, homemade) or ATP13A2 (SY3072, homemade [37] or A3361, Sigma) or with a mouse monoclonal antibody directed against mCherry (AB125096, Abcam) or GAPDH (G8795, Sigma). Proteins were detected using enhanced chemiluminescence (Supersignal^TM^ West Pico, LifeTechnologies) and the ChemiDoc^TM^ MP Imager (Bio-Rad). For detection of Ypk9p a monoclonal anti-FLAG antibody was used (F3165, Sigma) which was recognized by an anti-mouse antibody coupled to alkaline phosphatase (A4312, Sigma).

### Immunocytochemistry and fluorescence microscopy

For co-localization experiments, HeLa, MEF or Neuro2a cells were transiently co-transfected with fusion proteins of the different P5 isoforms (N- or C-terminal GFP- or mCherry-tagged) and different cellular markers (SERCA2b, Rab4/5/7/11, CLC-7 and Lamp1) in chamber slides. 48 h post transfection, cells were visualized with an Olympus IX81 fluorescence microscope using a 63x oil immersion objective as previously described in [37].

Immunocytochemistry experiments were performed on transiently transfected HeLa cells, cultured on gelatine-coated glass coverslips in 12-well plates. Cells were co-transfected with ATP13A1 or ATP13A2 in combination with the different cellular markers. 48 h post transfection, cells were washed in PBS, fixed in 4% paraformaldehyde in PBS for 20 min and subsequently permeabilized with 0.1% Triton X-100 in PBS for 4 min at room temperature. Blocking was performed for 1 h with 5% goat serum and 1% BSA in PBS. Primary homemade antibodies (ATP13A1, 1/500) or commercial antibodies (1/200; lamp1, Abcam ab25630; lamp2, Abcam ab25631; CD63, Exbio Praha 11-343-C100) and Alexa Fluor labeled secondary antibodies (1/2000) were diluted in 1% BSA in PBS. Glass coverslips were mounted on a microscope slide using FluorSafe^TM^ reagent (Calbiochem) and visualized with the Olympus IX81 fluorescence microscope or a Zeiss LSM510 confocal microscope. The Pearson’s correlation coefficient was determined with ImageJ software (NIH, Bethesda, Maryland, USA), using the JACoP plug-in [51].

For expression and localization of Ypk9p and ATP13A1-5 in yeast, constructs were inserted into the pAG425 vector containing an N- or C-terminal GFP fusion. *spf1*^*-*^ and *ypk9*^*-*^ deletion strains of *Saccharomyces cerevisiae* BY4741 (*MATa; his3Δ1; leu2Δ0; met15Δ0; ura3Δ0; yel039w*::*kanMX4 / MATa; his3Δ1; leu2Δ0; met15Δ0; ura3Δ0; yor291w*::*HIS3*) were transformed using the Li-acetate method and positive transformants were selected on synthetic complete dextrose agar plates (SCD-L; 2% glucose, 0.7% yeast nitrogen base, complete synthetic medium without leucine, 2% agar, 50 mM succinic acid-Tris pH = 5.5). Positive transformants were grown for 24 h on selective media conditions before dilution in 0.2% agar onto microscopy cover slides and subsequently visualized. Images were obtained using a Leica TCS SP5-X UV confocal laser scanning microscope with a 63x/1.20 numerical aperture water immersion objective (Leica Microsystems). GFP was excited at 488 nm and the emitting light was measured in the interval 500–533 nm. Brightfield and fluorescent images were processed using Leica software (Leica Microsystems). The contrast of the images was adjusted in a nonlinear manner to achieve qualitatively comparable signals for localization.

### Fluorescence protease protection (FPP) assay

HeLa cells were transiently transfected with N- or C-terminal GFP fusion proteins of ATP13A1-4 (WT or truncation mutants) and subjected to FPP as previously described [37, 52]. The minimal effective digitonin (Sigma, D141) concentration (20–40 μM), optimal time of digitonin treatment (30–90 s) and minimal effective trypsin concentration (100–150 μg/ml) were determined for each experiment in cells expressing GFP (cytosolic control protein) or ERO-GFP (ER luminal control protein) [37].

### Expression and purification of yeast Ypk9p

For overexpression of Ypk9p and Ypk9p D781N, *ypk9*^*-*^ cells were transformed with the overexpression plasmids (pRS423-pGAL10) using the Li-acetate method as described above. Both constructs encoded for a combined FLAG-RGS10xHis tag in the N-terminus of the proteins. Positive transformants were inoculated in 10 ml startup cultures (SCD-H; 2% glucose, 0.7% yeast nitrogen base, complete synthetic medium without histidine, 50 mM succinic acid-Tris pH = 5.5) overnight and transferred to 100 ml cultures grown for 12 hours at 30°C and 150 rpm (SCD-H). Samples were harvested by centrifugation and washed twice in sterile H_2_O before resuspension in 100 ml rich media (1% yeast extract, 2% bactopeptone supplemented with either 2% glucose (YPD) or 2% galactose (YPG)). Induction (YPG) or repression (YPD) of expression followed during growth for 16 hours at 30°C and 150 rpm. Finally, cells were harvested by centrifugation, washed twice in sterile H_2_O and frozen in liquid N_2_ and stored at -80°C.

Total membranes were purified by resuspending thawed cells at a 3:1 ratio (w/w) in lysis buffer (50 mM Tris, 1 mM EDTA, 0.6 M Sorbitol, 250 μl PMSF, 1 mM dithiothreitol (DTT), 2 μg/ml Pepstatin A, pH 7.5 with HCl). Glassbeads were added to half volume and the cells were lysed by vortexing for 16 cycles of 30 s with 30 s in between on ice. Samples were centrifuged for 15 min 1,000 g at 4°C and the supernatant was isolated and centrifuged again for 1 h at 160,000 g, 4°C. The pellet consisting of total membranes was resuspended in solubilization buffer and homogenized using a glass piston in a glass tube. Total protein was determined by Bradford assay and aliquots were frozen in liquid N_2_ and stored at -80°C. For purification of tagged Ypk9p and Ypk9p D781N proteins, total membranes were solubilized in solubilization buffer (20 mM MOPS, 130 mM KCl, 20% glycerol, 2 μg/ml pepstatin A, 1 mM DTT, 250 μM PMSF, pH 7.4 N-methyl-D-glucamine) supplemented with 5 mM imidazole and detergent *n*-Dodecyl β-D-maltoside (DDM) at a ratio of 2:1 with the total protein amount. Samples were incubated for 1 h with gentle rotation at 4°C. Following ultracentrifugation for 1 h 100,000 g at 4°C the supernatant was allowed to equilibrate for 2 h in a head over head rotator at 4°C with Ni-NTA (nitrilotriacetic acid) resin (Sigma) column, which had been pre-equilibrated with 4 column volumes (Cv) of binding buffer containing half the concentration of DDM used for solubilization. Washes were performed two times with 3x Cv of binding buffer supplemented with 10 mM imidazole and 0.05% DDM, and two times with 1.5x Cv of binding buffer supplemented with 20 mM imidazole and 0.05% DDM. Elution was performed using binding buffer supplemented with 300 mM imidazole and 0.005% DDM and collected in fractions of 250 μl. 12 μl of each fraction was used for SDS-PAGE following protein staining with Coomassie Blue. The protein concentration was determined by a Badford assay using γ-globulin as a standard.

### Autophosphorylation assay

Autophosphorylation of mCherry labeled ATP13A1-5 was performed as described in [37]. EP reaction buffer (17 mM Hepes pH 6.5, 160 mM KCl, 2 mM MgCl_2_, 1 mM DTT, 5 mM NaN_3_) was added to 40 μg of COS-8 membranes to a final volume of 95 μl. The reaction was initiated on ice by adding [γ-^32^P] ATP (2 μCi; 5.125 μM), 5.1 μM cold ATP. 400 μl stop solution (20% trichloroacetic acid, 10 mM phosphoric acid) was added after 5 min to stop the reaction. Samples were precipitated on ice for 20 min and centrifuged (20,000 g, 30 min, 4°C). Pellets were washed twice with 400 μl ice-cold stop solution and dissolved in sample buffer prior to acidic electrophoresis as previously described [12]. For measurements of Ypk9p and Ypk9p D781N activity the purified proteins were reactivated with a yeast polar lipid extract (Avanti) at a 1/3.55 protein to lipid ratio in reactivation buffer (50 mM Tris, 50 mM NaCl, 0.5% octyl β-D-glucopyranoside) by gently mixing protein and lipids dissolved in detergent following incubation for 30 min on ice. Autophosphorylation of the reactivated enzymes was performed essentially as described in [12] using the reaction buffer noted above. Samples were measured and quantified using scintillation counting in comparison to a ATP^32^ standard.

### Yeast complementation

Wild-type and knockout strains of *S*. *cerevisiae* BY4741 (WT—*MATa*: *his3Δ1; leu2Δ0; met15Δ0;ura3Δ0*; *spf1*^*-*^ *- MATa; his3Δ1; leu2Δ0; met15Δ0; ura3Δ0*: *yel039w*::*kanMX4* and *ypk9*^*-*^ *- MATa; his3Δ1; leu2Δ0; met15Δ0; ura3Δ0; yor291w*::*HIS3*) were transformed with the indicated constructs in the YEp351 vector using the Li-acetate method and positive transformants were selected on synthetic complete dextrose agar plates (SCD-L; 2% glucose, 0.7% yeast nitrogen base, complete synthetic medium without leucine, 2% agar, 50 mM succinic acid-Tris pH = 5.5). Transformed cells were grown overnight on SCD-L plates, suspended in sterile milliQ water and diluted to OD600 = 1. A dilution series of OD600 = 1, 0.1, and 0.01 was made with sterile milliQ water, and 5 μl of each dilution was spotted onto YPD plates (1% yeast extract, 2% bactopeptone, 2% galactose, 2% agar) or SCD-L plates containing caffeine or MnCl_2_ at indicated concentrations. Growth was recorded after 2 days at 30°C.

## Results

### Phylogenetic and functional relationship of the mammalian P5-type isoforms

To explore the relationship between the mammalian P5-type ATPase isoforms, we performed a phylogenetic analysis of 146 animal P5 ATPase sequences belonging to 22 different phyla across 43 individual organisms (including human ATP13A1-5). We included the yeast Spf1p and Ypk9p as more distant fungal representatives (S1 Table). The sequences were aligned and subsequently subjected to a phylogenetic analysis based on both the Bayesian inference and maximum likelihood methods, which provides superior statistics as compared to previous phylogenetic approaches that were used to compare the P5-ATPases in eukaryotes [44, 53]. The resulting phylogenetic model provides strong support for a strict division of the animal P5 ATPases into P5A and P5B groups (Figs 1 and 2 and S1 Fig), confirming previous work [44, 53].

All organisms tested had only a single P5A ATPase with one exception, namely *Salmo salar* (black arrow Fig 1). *S*. *salar* harbors two closely related P5A ATPase sequences, most likely reflecting a recent gene duplication in this species. The P5A sequences, which include the human ATP13A1 isoform, grouped together into a single monophyletic clade with Spf1p at its base (Figs 1 and 2).

The single yeast P5B isoform Ypk9p is found at the bottom of the P5B tree together with sequences of primitive animals such as the porifera *Amphimedon queenslandica* and the cnidarian *Mnemiopsis leidyi*, where also only one P5B isoform is found (red arrow Fig 1). This suggests that the fungal and early animal P5B sequences may be closely related. In contrast, all analyzed mammalian genomes contain four P5B ATPases, which belong to separate, isoform-specific clades that include related sequences from all other mammals. *Homo sapiens* ATP13A2, ATP13A3, ATP13A4, and ATP13A5 each belong to one of these isoform specific clades, which have been named accordingly (Figs 1 and 2). The isoform specific clades are a strong indication that the diversification into four isoforms occurred early in animal evolution, and/or that shortly following a gene duplication event a rapid diversification may have taken place. Interestingly, a fifth P5B clade (P5B_INV_ clade) appeared to be specific for invertebrates (protostomes) (Figs 1 and 2) and branched out at the base of the tree close to the ATP13A2 and ATP13A3 clades.

ATP13A2 and ATP13A3 presumably duplicated from a common P5B ancestor gene in the animal deuterostome lineage (yellow number 1, Figs 1 and 2), although the precise origin of the duplication is difficult to pinpoint (Fig 2, yellow number 1). The long evolutionary separation between ATP13A2 and ATP13A3 resulted in significant sequence diversity and two clearly separated clades. ATP13A2 may correspond more closely to invertebrate P5B orthologues than ATP13A3, since the ATP13A2 clade also includes some invertebrate sequences (green arrow Fig 1), while the ATP13A3 clade only includes vertebrate sequences. Remarkably, the ATP13A3-like sequences in the vertebrate lineage starting with early deuterostomes like echinoderms and primitive chordates (hemichordate and tunicate) (blue arrow Fig 1), appear to originate from a common ancestor of ATP13A3, despite that ATP13A2 may correspond more closely to the invertebrate P5Bs (Fig 1). With strong statistical support, the ATP13A4 and ATP13A5 clades evolved in higher vertebrates from two successive gene duplications of ATP13A3 (yellow numbers 2–3 in Figs 1 and 2), revealing a close relationship between the vertebrate isoforms ATP13A3-5. Only amniotes (reptiles, birds and mammals) contain two separate isoforms ATP13A4 and ATP13A5, while immediately before the split between ATP13A4 and ATP13A5, only a single ATP13A4-like sequence is recognized in the coelacanth *Latimeria chalumnae* and the amphibia *Xenopus laevis* (Figs 1 and 2). This suggests that a gene duplication of ATP13A4 took place before the rise of the reptiles (yellow number 3 in Figs 1 and 2), while ATP13A3 duplicated in the evolution of lobe-finned fish giving rise to the common progenitor of ATP13A4 and ATP13A5 (yellow number 2 in Figs 1 and 2).

In conclusion, the P5A ATPases did not diversify in animals, whereas the P5B ATPases diversified as the result of sequential gene duplications: one that occurred before the split between invertebrates and vertebrates, one that occurred after the split between ray-finned and lobe-finned fishes, and one between amphibia and reptilia evolution (Figs 1 and 2 and S1 Fig). This suggests that P5A ATPases may have a general and highly conserved function, whereas during vertebrate evolution P5B ATPases may have diversified to take up more specialized tissue-specific and/or subcellular functions.

An analysis of publicly available human microarray data (Genevestigator [54]) revealed that the P5A ATPase ATP13A1 is generally expressed in all cell types of *H*. *sapiens*, whereas the human P5B ATPases indeed show a more tissue specific expression pattern (S2 Fig). Although expressed in most tissues, ATP13A2 appears to be most highly expressed in the brain, which is also corroborated by a previous analysis of P5 ATPase gene expression in the mouse [42]. Also the *ATP13A3* gene displays a broad expression pattern, while *ATP13A4* and *ATP13A5* are highly expressed in specialized epithelial glandular cells. These are mainly associated with the lung (both ATP13A4 and ATP13A5—alveolar type 2 cells), mouth (ATP13A4—oral cavity, mucosa and gingiva) or nose (ATP13A5—nasal epithelium), but also sexual organs (ATP13A4 in mammary glands and ATP13A5 in testis epididymal corpus) (S2C and S2D Fig). In mice, the expression of the *ATP13A3* gene appears to be highly promoted during early embryonic development in contrast to mainly postnatal expression of ATP13A4 and ATP13A5 (S2B Fig).

### Mammalian P5-type proteins are *bona fide* P-type ATPases displaying autophosphorylation

**Fig 3:**
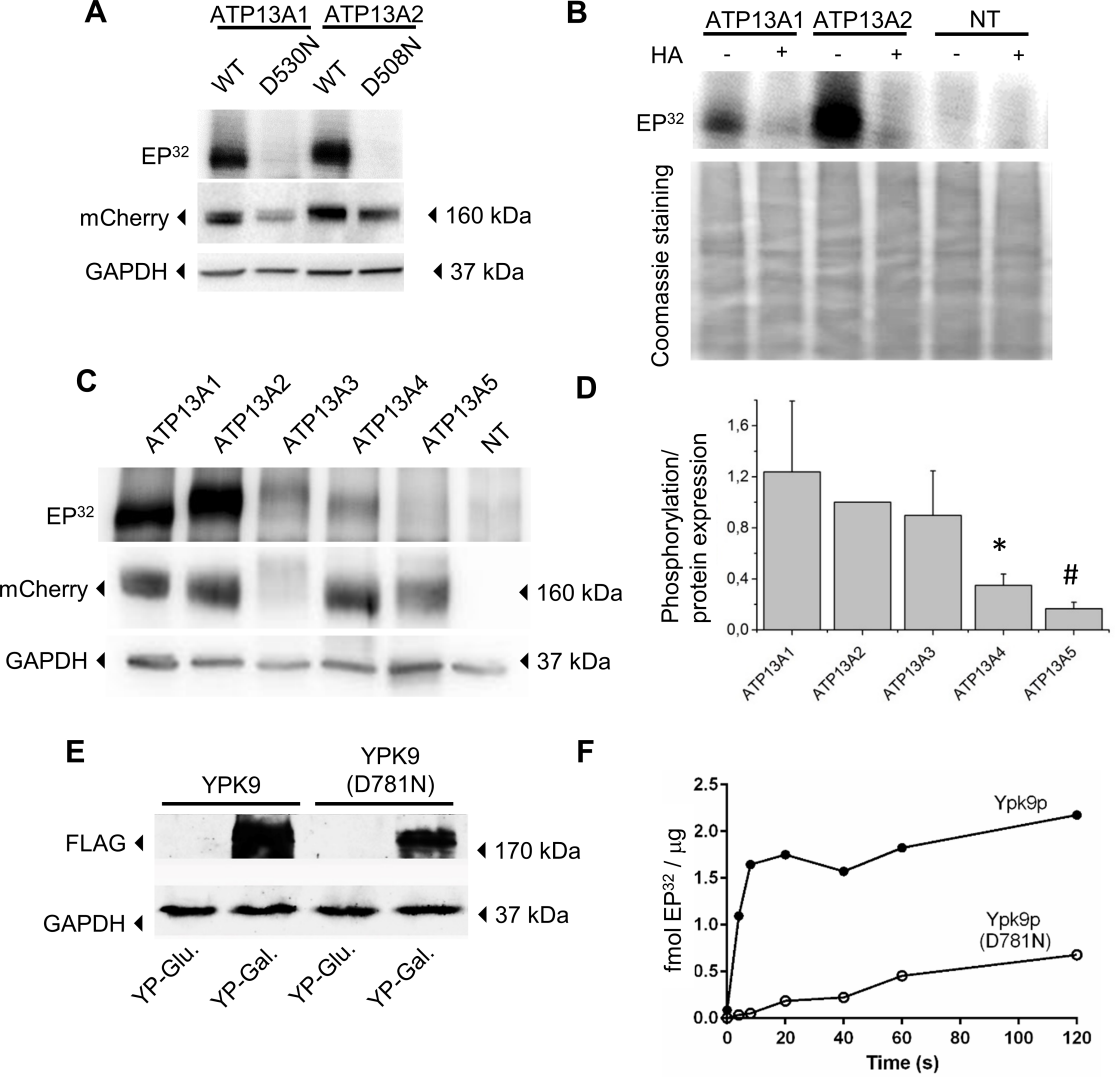
Auto-phosphorylation of the P5-type ATPases. **A-C.** Microsomal fractions of COS-8 cells, transiently overexpressing WT of dead mutants of the P5-isoforms (N-mCherry-tag) were analyzed on immunoblot or radiogram (EP^32^) to examine respectively expression levels and autophosphorylation. As a control, non-transfected cells (NT) were included. **A.** Immunoblot with mCherry antibody shows overexpression for ATP13A1 and ATP13A2 WT and dead mutants. GAPDH levels were determined as loading control. The radiogram (EP^32^) demonstrates phosphorylation for WT ATP13A1 and ATP13A2, but not for dead mutants. **B.** Radiogram indicates that ATP13A1 and ATP13A2 are both sensitive towards hydroxylamine (HA), a hallmark of P-type ATPases. HA quenches the phosphorylated Asp residues, but leaves phosphorylated Thr, Ser or Tyr residues intact. The lower panel shows a coomassie stained gel that depicts the equal loading for all lanes in the upper panel. **C.** Immunoblot and radiogram of the autophosphorylated intermediates (EP^32^) of the five P5 isoforms demonstrate overexpression of all isoforms, whereas phosphorylation was only seen for ATP13A1-4. **D.** Graph showing the autophosphorylated levels (EP^32^ panel C) normalized to the relative expression levels of each isoform (mCherry immunoblot panel C). Results indicate strongest autophosphorylation for ATP13A1, ATP13A2 and ATP13A3. ATP13A4 displays low autophosphorylation levels, while ATP13A5 levels are negligible. Data are represented as average ± SD (n = 4). *, P ≤ 0.05 *versus* ATP13A1 and ATP13A2; #, P ≤ 0.05 *versus* ATP13A1, ATP13A2 and ATP13A3 (one-way ANOVA, Bonferroni post-hoc). **E.** Immunoblot demonstrating the expression of the N-terminal 10xHis-FLAG tagged versions of the yeast Ypk9p and Ypk9p (D781N) in yeast total membrane fractions. Antibody against the FLAG part of the tag was used to visualize protein expression and GAPDH was used as a loading control. The wildtype but not catalytic dead protein was able to complement loss of the native *YPK9* gene (Fig 6B). Both Ypk9p variants were successfully purified using the 10xHis part of the tag (see S3 Fig). Proteins are expressed from the galactose inducible promotor when grown in rich media supplemented with galactose (YP-Gal) while glucose repress expression (YP-Glu). **F.** Quantification of Ypk9p autophosphorylation using scintillation counting, normalized to μg of purified Ypk9p. Purified Ypk9p was able to undergo autophosphorylation, which was abolished by mutating the Asp residue in the catalytic motif (D781N).

We previously demonstrated that in isolated membrane fractions hATP13A2 undergoes autophosphorylation on the acceptor residue D508 [37]. This aspartyl phosphorylation is a hallmark feature of P-type ATPase functionality. We now explored whether other P5A- and P5B-type ATPases may undergo autophosphorylation to compare the relationship of P5-type ATPases at the biochemical level. We confirmed that ATP13A1-5 isoforms contain the typical P-type ATPase sequence motifs for coupled transport, which involves catalytic autophosphorylation on a conserved Asp residue [2]. While ATP13A2 and ATP13A3 cDNA was already available, we first cloned the cDNA of the ATP13A1, 4 and 5 isoforms from a mouse brain mRNA preparation. We then assessed the autophosphorylation activity of N-terminal mCherry labeled P5 constructs in a COS-8 overexpression system (Fig 3) [37]. Like ATP13A2, we now showed that also the mammalian P5A-type ATPase ATP13A1 is autophosphorylated (EP^32^, Fig 3A–3D). No traces of a phospho-intermediate were observed for the ATP13A1 D530N mutant, which cannot be solely attributed to the approximately three-fold lower expression levels of the mutant *versus* WT (mCherry immunostaining, Fig 3A). The mATP13A1 and hATP13A2 phosphoproteins were highly sensitive towards hydroxylamine, which is a characteristic of aspartyl phosphorylation in P-type ATPases (Fig 3B) [55]. Hydroxylamine chemically attacks phosphorylated Asp residues, while leaving phosphorylated Ser, Thr or Tyr residues intact. Thus, the hydroxylamine sensitivity of the phosphorylated bands, together with the loss of phosphorylation in the D530N mutant prove that autophosphorylation of ATP13A1 takes place on the conserved D530 in the P-type ATPase signature motif.

We also observed autophosphorylation of hATP13A3 and mATP13A4 (Fig 3C and 3D), although only in four out of eight independent membrane preparations, which limited further biochemical analysis with hydroxylamine. Several parameters such as cell density, passage number, degree of transient transfection, availability of the substrate and cellular stress conditions may possibly contribute to this variable behavior. In none of the eight independent preparations, we detected autophosphorylation of ATP13A5, despite sufficiently high expression (Fig 3C).

Compared to the mammalian P5-type ATPases also the two *S*. *cerevisiae* P5-type ATPases Spf1p and Ypk9p contain the highly conserved P-type ATPase sequence motifs required for transport. Autophosphorylation was previously reported for Spf1p, which takes place on the Asp487 residue in the autophosphorylation motif [56]. To produce Ypk9p protein for biochemical assays we purified 10xHis-FLAG tagged versions of Ypk9p and the catalytic dead mutant Ypk9p (D781N) from overexpression in yeast (Fig 3E and 3F, and S3 Fig) and could demonstrate that Ypk9p undergoes autophosphorylation, which is impaired by a mutation of the catalytic Asp781 residue in the P-type motif for autophosphorylation (Fig 3F).

In conclusion, we have demonstrated autophosphorylation on the catalytic Asp residue of ATP13A1 and ATP13A2, while we observed indications for autophosphorylation of ATP13A3 and ATP13A4. Thus, several mammalian and yeast P5A- and P5B-type ATPase isoforms display the typical characteristics for autophosphorylation, which may represent a hallmark of P5-type ATPases.

### ATP13A1 is an endoplasmic reticulum-localized pump with 12 transmembrane helices

**Fig 4:**
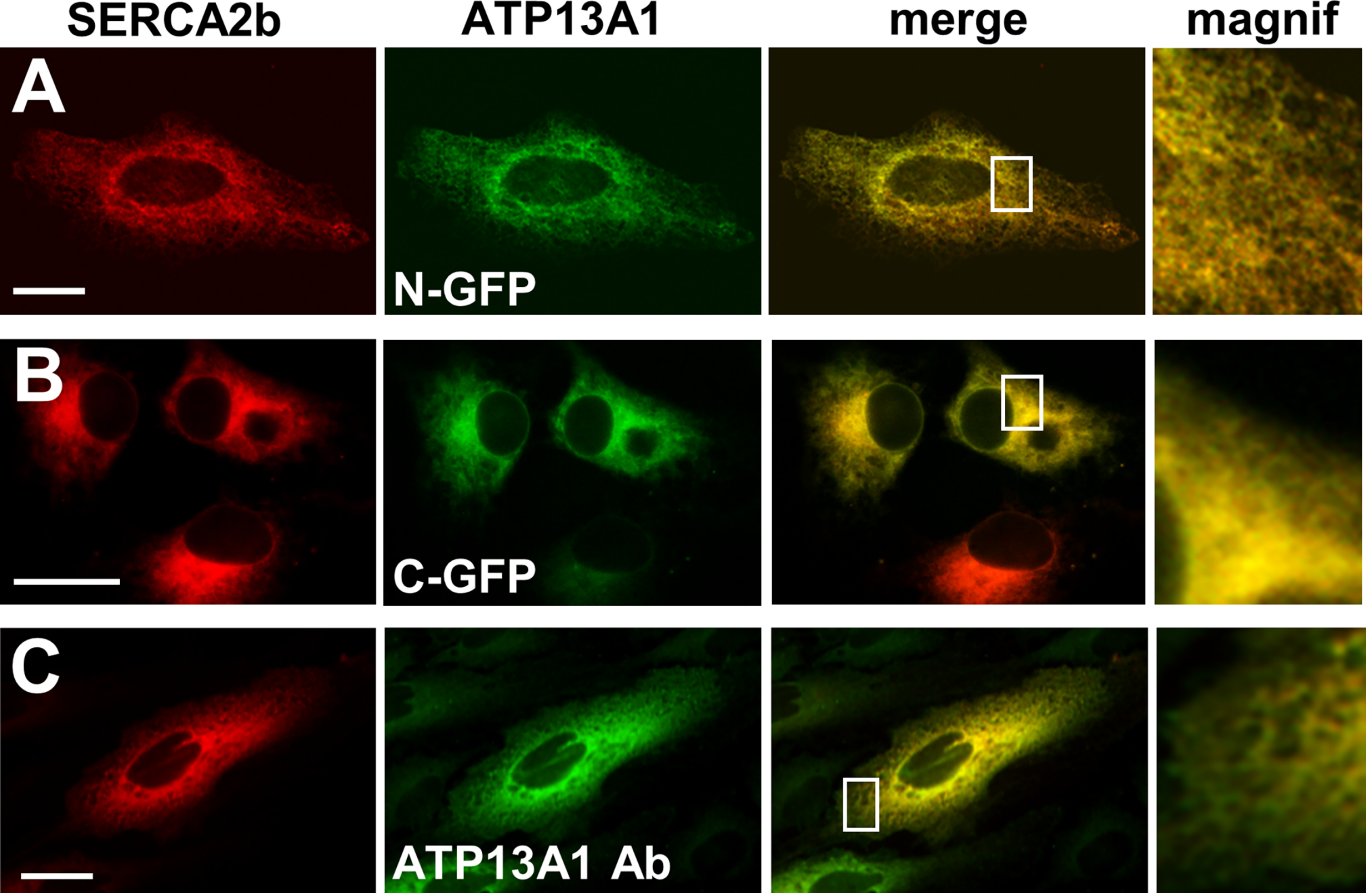
ATP13A1 localizes to the endoplasmic reticulum. HeLa cells were transiently co-transfected with ATP13A1 wildtype (no tag, N- or C-terminal GFP-tag) or dead mutant (N-terminal GFP-tag) and SERCA2b-mCherry, an ER resident. **A-C.** ATP13A1 with N- (**A**) or C-terminal (**B**) GFP-tag or unlabeled ATP13A1 (**C**) co-localizes with SERCA2b. Unlabeled ATP13A1 was detected via immunocytochemistry with homemade ATP13A1 antibody (S4 Fig) and Alexa Fluor 488. Scale bar represents 20 μm.

**Fig 5:**
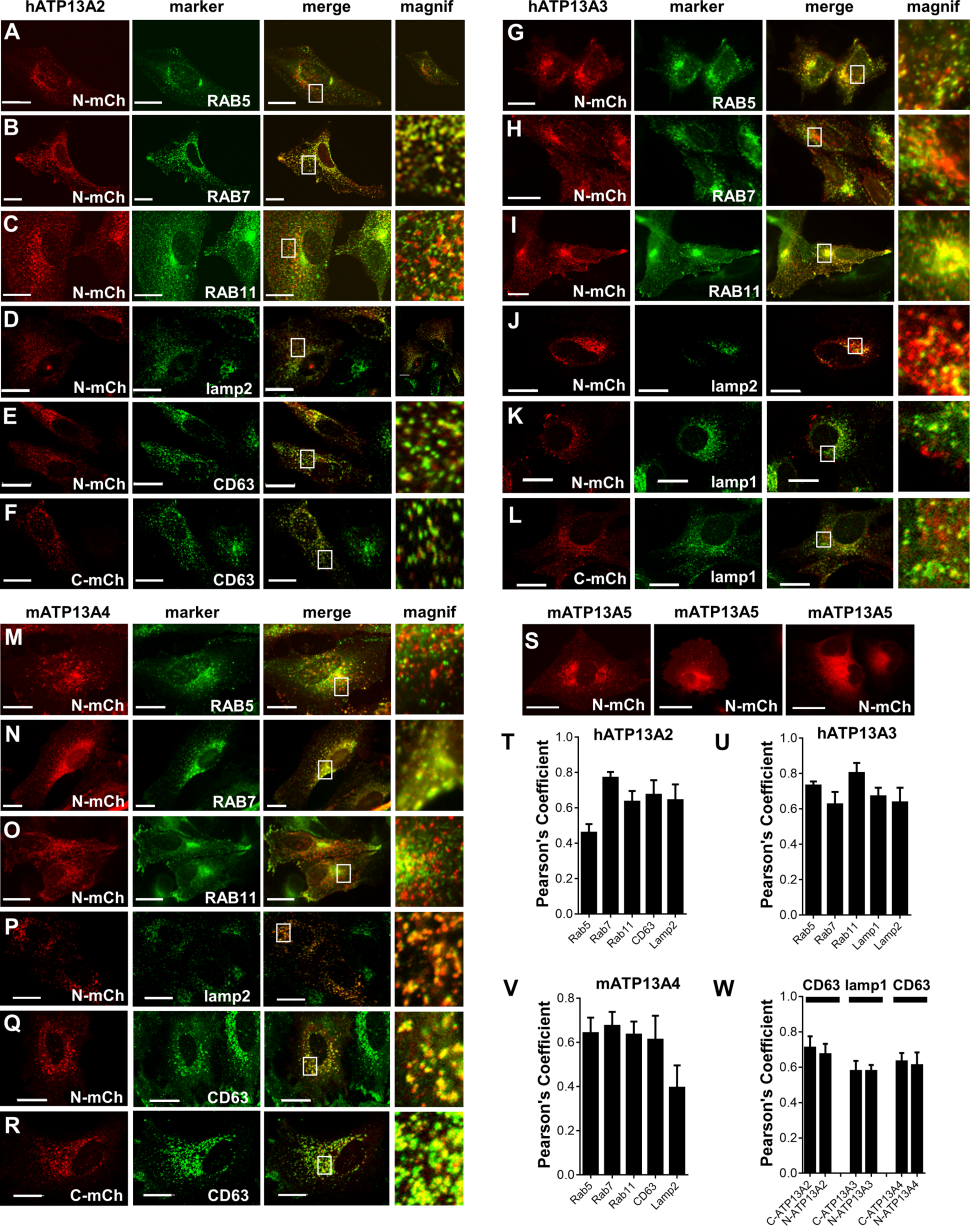
ATP13A2-4 localize to the endo-/lysosomal system. HeLa cells were transiently co-transfected with hATP13A2, hATP13A3, mATP13A4 or mATP13A5 (N- or C-terminal mCherry-tag, N-mCh and C-mCh, respectively). Scale bar represents 20 μm. **A-C.** Co-localization of hATP13A2 (N-terminal mCherry-tag) with various GFP-labeled markers, such as the early endosomal marker RAB5 (A), the late endo-/lysosomal marker RAB7 (B) and RAB11, a marker for the recycling endosomes (C). **D-F.** Immunocytochemistry of cells overexpressing N- or C-terminal mCherry labeled hATP13A2 with the endogenous markers of late endosomes (CD63, E-F) and lysosomes (lamp2, D). **G-I.** Co-localization of hATP13A3 with GFP-labeled RAB5 (G), RAB7 (H) and RAB11 (I). **J-L.** Immunocytochemistry of cells overexpressing N- or C-terminal mCherry labeled hATP13A3 with the endogenous markers of late endo-/lysosomes lamp1 (K, L) and of lysosomes lamp2 (J). **M-O.** Co-localization of hATP13A4 with GFP-labeled RAB5 (M), RAB7 (N) and RAB11 (O). **P-R.** Immunocytochemistry of cells overexpressing N- or C-terminal mCherry labeled hATP13A4 with the endogenous markers of late endosomes (CD63, Q-R) and lysosomes (lamp2, P). **S.** mATP13A5 overexpression is too weak for fluorescence microscopy. When a fluorescent signal is observed, an endosomal-like pattern was seen in 80% of the observations (two left panels), whereas in other images a reticular pattern was observed (right panel). **T-W.** Bar graphs depict Pearson’s coefficients (PCC) of ATP13A2-5 isoforms with various endosomal markers.

The different P5A and P5B isoforms in vertebrates may not only fulfill tissue specific functions (S2 Fig), but may also be targeted to various subcellular compartments where they may exert dedicated cellular functions. To explore this possibility, we compared the subcellular distribution of the five mammalian isoforms ATP13A1-5 in several cell types (Figs 4 and 5). We generated GFP- and mCherry-tagged constructs for all P5B isoforms, which were co-expressed in HeLa, Neuro2a or MEF cells with various tagged marker proteins for confocal microscopy studies. The data below (Figs 4 and 5) represent images of HeLa cells, but we confirmed a similar targeting pattern in Neuro2a and MEF cells for all constructs (data not shown).

mATP13A1-GFP (Fig 4A, WT and D530N mutant) co-localized well with SERCA2b, a well-characterized endoplasmic reticulum (ER) P-type ATPase [57] (Pearson’s correlation coefficient (PCC) of 0.827 ± 0.079). In contrast, we observed no co-localization with the Golgi/Secretory Pathway Ca^2+^ ATPase or various endosomal markers (RAB4, 5, 7 and 11) (data not shown). We also tested whether the position or presence of a fluorescent tag may affect the localization. Both N- and C-terminal GFP fusion constructs were found in the ER (Fig 4A and 4B) and we confirmed with a homemade polyclonal antibody raised against mATP13A1 (S4 Fig) that also the unlabeled mATP13A1 remains in the ER (Fig 4C). Also, the catalytically inactive mutant of mATP13A1 (D530N) colocalizes with SERCA2b, indicating that the transport activity doesn’t influence the localization (S5A Fig). Although we confirmed that mATP13A1 is expressed at the mRNA level, we were unable to detect the endogenous mATP13A1 protein, possibly because of the low abundance of the endogenous protein or the low affinity of the antibody.

Compared to most other mammalian P-type ATPases that typically contain 10 TM helices, topology predictions suggest that on top of the typical 10 TM helices, ATP13A1 may contain an additional N-terminal hairpin [53]. We experimentally confirmed the existence of this N-terminal hairpin by a fluorescence protease protection (FPP) assay [52], which is a widely accepted method to study topology, which was used before to establish the topology of ATP13A2 [37]. We performed an FPP assay on full-length ATP13A1 and N-terminal constructs fused to GFP at various positions (S6 Fig). The protection of the GFP tag against trypsin cleavage indicates a luminal position of the GFP tag (as with the ER-localized ERO-GFP), while the quenching of the signal reflects a cytosolic position (as with the cytosolic GFP). Our data show that ATP13A1 contains an N-terminal hairpin in addition to the 10 predicted TM helices that are conserved amongst P-type ATPases. It is unlikely that the GFP tag may disrupt the topology, since N- and C-labeled ATP13A1 proteins remain active (Fig 3A, and also S8 Fig as explained below).

Thus, we demonstrate that mATP13A1, the mammalian P5A representative, is an ER resident protein that most likely contains 12 TM helices, which would be in line with earlier topology predictions [53].

### Mammalian P5B ATPases target to late endo-/lysosomes and contain an even number of TM helices

For the localization of the P5B-ATPases, we turned to N-terminal mCherry labeled ATP13A2-5 constructs. No clear signs of organelle abnormalities or cell toxicity were found as a consequence of overexpressing the P5B isoforms. We first confirmed that the localization of mCherry labeled ATP13A2 corresponds with previous reports (Fig 5T) [11, 19, 37]. Indeed, we observed co-localization with GFP-labeled markers RAB7 (Fig 5B, late endo-/lysosomes), while the overlap with RAB5 (Fig 5A, early endosomes) or RAB11 (Fig 5C, recycling endosomes) was lower. Also, the unlabeled catalytically inactive mutant of hATP13A2 (D508N) is found in the late endo-/lysosomes indicating that the transport activity or tag has no impact on the localization (S5B Fig). Via immunocytochemistry we confirmed that ATP13A2 co-localized well with endogenous markers of the late endosomal compartment, CD63 (Fig 5E and 5F), and lysosomes, lamp2 (Fig 5D), which is consistent with previous reports [11, 19, 37]. Importantly, we also observed a similar distribution for the N- or C-terminal fusion proteins of ATP13A2, indicating that the position of the tag has no effect on the localization (Fig 5E, 5F and 5W), as highlighted before [37].

The mCherry labeled hATP13A3 constructs overlapped to some degree with several GFP-labeled markers of the early (Fig 5G) and late endo-/lysosomes (Fig 5H), but the best co-localization was observed with markers of the recycling endosomes (Fig 5I). We confirmed by immunolocalization a lower overlap of ATP13A3 with endogenous markers of the late endosomes (Fig 5K and 5L) and lysosomes (Fig 5J). Finally, the localization of the N- or C-terminal labeled ATP13A3 was similar (Fig 5K, 5L and 5W), and also GFP-labeled ATP13A3 constructs displayed a comparable subcellular distribution (S7D and S7E Fig). Together, our results indicate that in the overexpression system ATP13A3 is mainly targeted to the recycling endosomes, and to a lesser extent to the late endo-/lysosomal compartment (Fig 5U).

For ATP13A4, we observed a broader overlap with various GFP labeled markers of the endosomal compartment (Fig 5V): RAB5 (Fig 5M), RAB7 (Fig 5N) and RAB11 (Fig 5O). Via immunolocalization with endogenous markers, we found a similar degree of overlap with CD63 (Fig 5Q and 5R) as for RAB7 (Fig 5N), while the overlap with lysosomes is lower (Fig 5P). Little effect of the position of the tag is observed (Fig 5Q, 5R and 5W), and also the GFP-labeled ATP13A4 constructs display a similar subcellular distribution (S7B and S7C Fig).

Unfortunately, we were unable to unambiguously determine the localization of ATP13A5, although we tested various tags on mATP13A5 (V5, GFP and mCherry either on the N- or C-terminus) and expression in different cell types (COS-8, MEF, HeLa and Neuro2a) (data not shown). The low percentage of fluorescently labeled HeLa cells mainly displayed a vesicular pattern (Fig 5S), but co-expression with endosomal markers failed. Moreover, we often observed a more reticular ER-like distribution pattern for mATP13A5 in the transfected population, which could indicate that the expressed protein had problems leaving the ER (different panels of Fig 5S).

Earlier topology predictions indicated that all P5B ATPases contain an unusual N-terminal membrane segment (Ma) that may represent an extra TM region at the N-terminus, resulting in a total number of 11 TM helices [53]. However, we recently demonstrated with an FPP assay that hATP13A2 consists of an even number of TM regions and that the unusual N-terminal hydrophobic segment (Ma) does not span the membrane. Thus, experimental verification of the topology predictions is important [37] (S7A Fig). Here, we established that like ATP13A2, also ATP13A3 and ATP13A4 display an even number of TM helices, which further refutes the earlier P5B topology predictions [53]. Indeed, both the N- and C-termini of GFP-labeled hATP13A3 (S7B and S7C Fig) and mATP13A4 (S7D and S7E Fig) are cytosolic, since the fluorescent tag is sensitive to trypsin degradation. Note that the N- or C-terminal addition of fluorescent tags did not prevent the exit of ATP13A2-4 from the ER. Also the autophophosphorylation activity of N-terminal labeled protein remains intact (Fig 3C and 3D). Together, this indicates that the fusion proteins are properly folded and that the topology is intact. Based on these FPP data and the strong conservation of the N-terminal Ma segment in all P5B-ATPases, we expect that like ATP13A2, also other P5B-ATPases contain an N-terminal membrane associated fragment.

In conclusion, our data support the division of mammalian P5 type ATPases into a P5A and P5B group. mATP13A1 represents an ER resident protein with most likely 12 TM helices, while ATP13A2-4 reside mainly in overlapping compartments of the endosomal system and present a membrane topology that differs from ATP13A1.

### The mammalian P5A isoform ATP13A1 complements the yeast P5A deletion mutant

**Fig 6:**
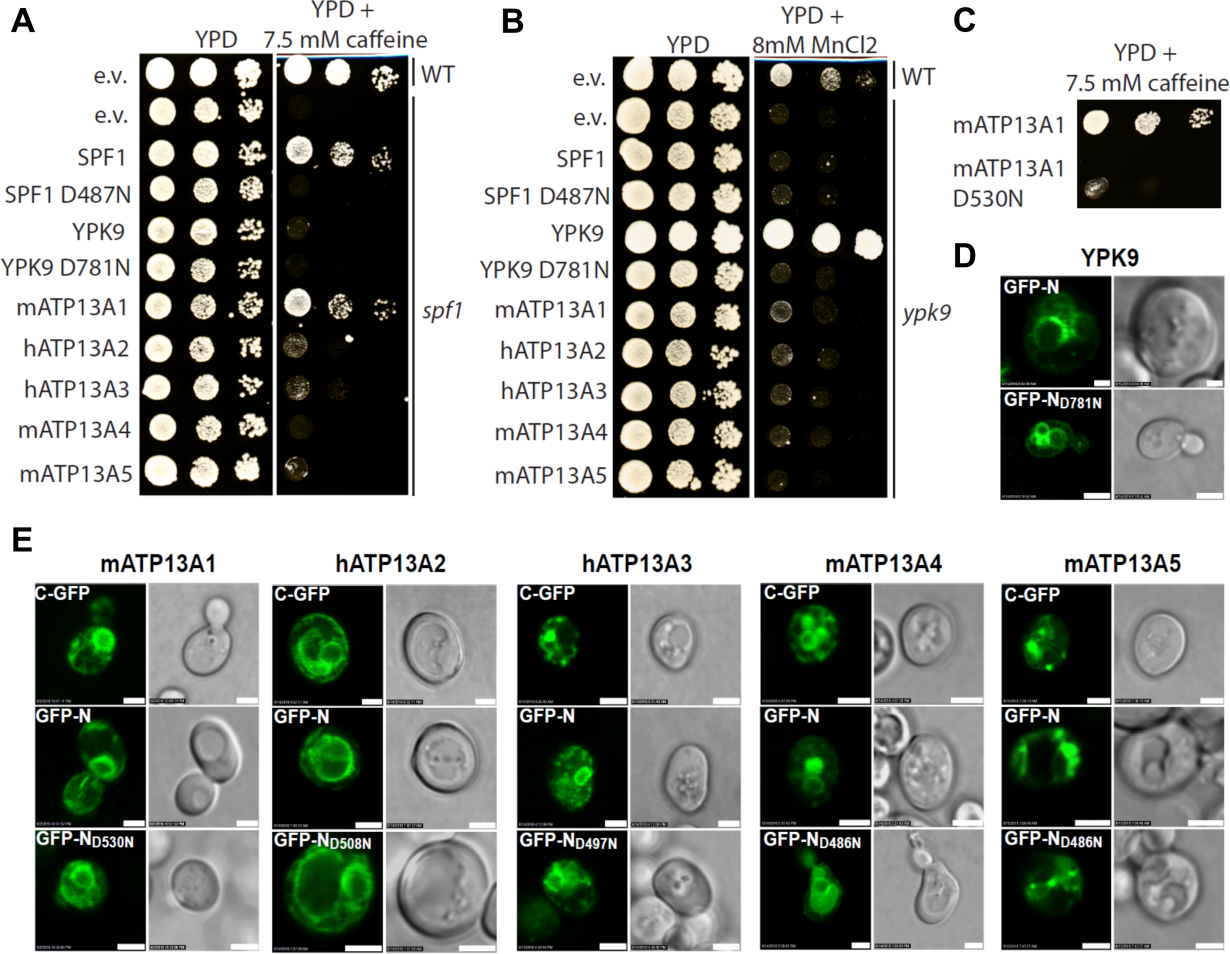
Genetic complementation in yeast with mammalian P5-type ATPases. The genome of *S*. *cerevisiae* contains a single P5A ATPase gene (*SPF1*) and a single P5B ATPase gene (*YPK9*). **A.** Gene knockout of *SPF1* results in increased sensitivity to caffeine which can be rescued by expression of untagged Spf1p and mATP13A1 respectively, but not by the untagged catalytically dead SPF1 D487N or by untagged mammalian ATP13A2-5. (e.v.–empty vector) **B.** Deletion of *ypk9*^*-*^ results in increased sensitivity to MnCl_2_, which can be rescued by expression of untagged Ypk9p, but not by the untagged and catalytically dead Ypk9p D781N. None of the untagged mammalian P5 ATPase genes showed rescue of *ypk9*^*-*^ (not shown). **C.** ATP13A1 complements the caffeine phenotype of the *spf1*^*-*^ knockout strain, whereas the catalytically dead mutant D530N fails to complement. **D.** Expression of Ypk9p and catalytically dead Ypk9p D781N proteins was confirmed by both N-terminal GFP fusion constructs. Ypk9p tagged with GFP show vacuolar localization. **E.** Expression of mammalian ATP13A1-5 proteins was similarly confirmed by N- and C-terminal GFP fusion constructs. N- or C-terminal positioning of GFP showed no apparent difference in localization patterns. ATP13A1 shows ER localization and is absent in vacuoles, ATP13A2 and ATP13A4 show vacuolar localization, ATP13A3 and ATP13A5 show localization to cytosolic spots which could represent a pre-vacuolar compartment or early endosomes. Scale bars represent 2,5 μm.

Next, we assessed the functional and physiological relationship between yeast and mammalian P5-type ATPases via a yeast complementation assay. Similar to the re-introduction of *SPF1*, expression of the human P5A isoform ATP13A1 rescued the impaired growth of the *spf1*^*-*^ deletion strain on caffeine (Fig 6A). Autophosphorylation deficient D/N mutants of Spf1p (Fig 6A) and ATP13A1 (Fig 6C) were not functional. We further demonstrate that the N-GFP and C-GFP labeled ATP13A1 constructs are targeted to the ER (Fig 6E), like in mammalian cells (Fig 4), and functionally complement the yeast *spf1*^*-*^ phenotype (S8 Fig). This strongly indicates that the addition of fluorescent tags does not disrupt the function, topology or subcellular localization of ATP13A1. The complementation by an animal P5A ATPase gene of a fungal orthologue points to a highly conserved function of P5A ATPases in the ER. In contrast, none of the four human P5B ATPases, nor Ypk9p, were able to complement the caffeine sensitivity of *spf1*^*-*^ cells (Fig 6A).

It was already reported that ATP13A2 fails to complement the *ypk9*^*-*^ deletion strain [5, 10]. Moreover, none of the four mammalian P5B ATPases were able to rescue the Mn^2+^ sensitivity of the *ypk9*^*-*^ strain (Fig 6B). This deficiency may be due to lack of expression or cellular mis-localization in the heterologous expression system. To investigate this, GFP-labeled Ypk9p and ATP13A1-5 were expressed in yeast and we observed that Ypk9p-, ATP13A2- and ATP13A4-GFP constructs were clearly targeted to the vacuole (the counterpart of the mammalian lysosomal compartment) (Fig 6D and 6E). The failure of the mammalian P5B ATPases to complement the yeast *ypk9*^*-*^ deletion strain, despite the fact that they were expressed and targeted to the same membrane system as Ypk9p, may support the view that the animal and yeast P5B ATPases may have evolved different cellular functions or may be regulated differently.

## Discussion

In this study, we compared the properties and relationship of the five mammalian P5-type ATPase gene products, ATP13A1-5, for which the function is not yet established.

Our phylogenetic analysis subdivides the animal P5-type ATPase into a P5A (ATP13A1) and P5B (ATP13A2-5) group, which may reflect fundamental differences in cellular function. The distinction between P5A and P5B already appeared early in eukaryote evolution [44, 53] and here we provide further support for this clear distinction based on differences in subcellular localization, membrane topology and functional complementation in yeast. The presence of charged *versus* uncharged residues in the M4 helix at the position of the putative substrate binding site (PP(E/D)xPx(E/D) *versus* PP(A/V)xP(A/V)x motif in P5A and P5B, respectively) possibly indicates that members of the two groups transport different substrates [44, 53]. All animal species contain exactly one P5A member, whereas additional P5B isoforms appeared in vertebrate evolution (ATP13A2-5) after successive gene duplications, with ATP13A2 being the oldest representative. An important question is whether these various P5B isoforms may fulfill similar cellular roles. The conserved putative substrate-binding motif in ATP13A2-5 indicates that the vertebrate P5B isoforms may transport the same or a closely related ligand. Instead, based on their expression pattern and subcellular localization, the various P5B-ATPases may exert tissue-specific functions and/or may fulfill overlapping roles in sub-compartments of the endosomal system. The P5B isoform diversity in higher vertebrates seems to be particularly relevant in the brain, where all isoforms are co-expressed. Also, the dysfunction of ATP13A2 and ATP13A4 are linked to various neurological disorders.

To get closer to answering these questions, we cloned several mammalian P5B isoforms for the first time, generated molecular tools and protocols for their study and optimized the first biochemical assays, which will facilitate further research. So far, we observed autophosphorylation of the ATP13A1-4 isoforms without adding a particular ligand to stimulate activity. This may indicate that the transported ligand of the P5 ATPases is membrane associated or that the P5 ATPases undergo autophosphorylation prior to the binding of their substrate, similar to what is observed for the P4-type ATPases [1, 4].

### P5A ATPases exert a highly conserved ER function

We show with various complementary methods using tagged and untagged protein that the mammalian ATP13A1 isoform is an ER resident protein. Irrespective of the tag and species (mammalian *versus* yeast cells) we observed a clear reticular ER distribution for ATP13A1. This pattern is not merely a result of improper folding of ATP13A1 due to the overexpression. Indeed, we demonstrated via biochemical and complementation assays that ATP13A1 remains functional. The ER distribution for ATP13A1 is similar to the localization of other P5A orthologues in *Saccharomyces pombe* [58] and *Arabidopsis thaliana* [59]. The *S*. *cerevisiae* orthologue Spf1p resides in the ER membrane [17, 60], but may also be found in *cis*-Golgi membrane fractions [61]. The P5A ATPases appear to play an essential role in the ER, since all animal genomes typically contain a single highly conserved P5A ATPase gene. Moreover, like the barley HvP5A1 isoform [12], the mammalian ATP13A1 isoform complements loss of *SPF1* in yeast showing that P5A-type ATPases exert identical or overlapping ER functions across different species. Thus, our results demonstrate that the already established yeast *spf1*^*-*^ phenotype will provide key information on the physiological role of ATP13A1 in mammalian cells, whereas ATP13A1 functionality can be studied in yeast.

The exact role of the P5A ATPases in the ER remains unidentified, but several ER-related properties are affected by the loss of *SPF1* in yeast. Loss of the *SPF1* gene influences the unfolded protein response [62], the insertion of tail-anchored or transmembrane proteins into the membrane and it controls the topology of integral membrane proteins [63–65]. Furthermore, *spf1* affects the glycosylation of secreted proteins [60, 66–69], the translocation of secretory proteins across the ER membrane [66, 67, 70, 71] and vesicle trafficking [66, 67, 70–72]. Finally, deletion of *spf1* impairs the biosynthesis and transport of sterols [17, 65, 73] and may be implicated in Ca^2+^ homeostasis [3, 12, 73, 74]. Our complementation results indicate that ATP13A1 may play a similar physiological role as Spf1p and may therefore also be involved in these processes in mammalian cells.

### The ATP13A2-5 isoforms may fulfill partially overlapping functions in the endo-/lysosomes

Our results show that the ATP13A2-4 isoforms are targeted to overlapping compartments of the endosomal system, which differs from the ER localization of P5A ATPases. The late endo-/lysosomal localization of ATP13A2 in mammalian cells [19] and the targeting of Ypk9p in *S*. *cerevisiae* to the vacuole, the equivalent organelle of the lysosomes in yeast were already described [5]. Further in line with a late endo-/lysosomal function, ATP13A2 deficiency in fibroblasts derived from Kufor Rakeb disease patients [16] causes an increase in size and number of lysosomes, diminished lysosomal-mediated clearance of autophagosomes and impaired lysosomal degradation [75]. Whether loss of ATP13A3-5 function may lead to a similar endo-/lysosomal phenotype needs further investigation, but the presence of multiple P5B isoforms in overlapping endosomal sub-compartments indicates that the P5B ATPases may fulfill partially redundant functions, although each isoform displays a slightly different endosomal targeting. ATP13A2 is predominantly found in late endo-/lysosomes, ATP13A3 in recycling endosomes and ATP13A4 displays a broader distribution.

The targeting to the endosomes may not be general for all P5B-ATPase orthologues. One of the *C*. *elegans* P5B orthologues CATP-5 is associated with the plasma membrane [76]. Also, a previous study reported the targeting of ATP13A4 to the ER [39], although here we clearly observe an endosomal distribution of ATP13A4, indicating that targeting motifs for the endocytic compartment must be present. The discrepancy for ATP13A4 may relate to differences in cell type and expression of co-factors important for the targeting or the level of overexpression. Unfortunately, we were unable to observe reproducible expression, localization or activity of ATP13A5 in different cell models. We verified that the cDNA sequence corresponds to the reported sequence in the gene database dbEST (accession number Q3TYU2.2) and we tested two independent cDNA constructs obtained by two commercial vendors. Since ATP13A5 displays a more restricted tissue distribution than other P5 ATPases [42], the stable ATP13A5 expression and subcellular targeting may require a specific cell type, expressing auxiliary proteins or containing essential co-factors or substrates.

The subcellular localization of tagged ATP13A1-5 proteins in an overexpression system may not necessarily reflect the localization of the endogenous proteins. However, we could exclude that the tag or the protein activity interfered with the targeting as we observed a similar subcellular distribution for various N- and C-terminal fusion proteins and inactive proteins, and no signs of abnormalities in the endosomal system were found. Still, the overexpression may saturate subcompartments of the endo-/lysosomal system that may result in a slightly broader endosomal distribution as compared to the endogenous protein, which is reflected by our PCC values. This was previously reported for ATP13A2, but the differences between endogenous and overexpressed ATP13A2 were subtle [11] and the conclusion that ATP13A2 is targeted to late endo-/lysosomes remains valid. This suggests that the overexpression system provides a good approximation to determine the intracellular targeting, but future studies with high quality antibodies will be required to confirm the localization on the endogenous proteins.

Based on our FPP analysis, we can conclude that the topology of the N-terminal regions of ATP13A2-4 is highly similar, but different from ATP13A1, as our FPP analysis only deals with the topology of the N-terminus, and other TM regions were not verified by this approach. However, sequence alignments and topology predictions of the P5-type ATPases already indicated that all P5 isoforms have 10 TM helices the core protein in common with other P2, P3 and P4-type ATPases [53]. The P5 ATPases only differ at their N-terminus by harboring an additional hairpin motif (P5A) or a conserved hydrophobic region Ma that is not spanning the membrane (P5B). For all P5B ATPases, Ma is similar in size and composition and contains a highly conserved Gly residue. This Gly residue may introduce a kink in the membrane associated region in order to keep the hydrophobic segment at the cytosolic membrane leaflet, as was demonstrated for ATP13A2 [37].

As none of the mammalian P5B isoforms did complement the Mn^2+^ toxicity phenotype of the yeast *ypk9*^*-*^ deletion strain, we were unable to draw any conclusions on the functional relationship between the animal and fungal proteins. The mammalian P5B isoforms may not be functional in yeast due to the lack of regulatory mechanisms, post-translational modifications or dependence on other co-factors. However, this negative result may also indicate that the substrate specificity of the animal P5B ATPases is different from the yeast P5B, questioning whether yeast is a good host to evaluate the mammalian P5B function. The evolution of the P5B-type ATPases is marked by multiple parallel gene duplication/deletion events and rapid diversification, making it difficult to unambiguously assess the phylogenetic and functional relationship between vertebrate ATP13A2 and ATP13A3, or between yeast, vertebrate and invertebrate P5B orthologues. To assess the functional relationship in the future, complementation experiments may be conducted in animal model organisms, like *C*. *elegans*, *D*. *melanogaster* or *D*. *rerio*. It is clear from our phylogenetic analysis that invertebrate P5B isoforms group in a different clade than the vertebrate isoforms, possibly indicating that their functions may have diverged. Therefore, vertebrate models may be required to study the relationship of the four mammalian P5B isoforms.

In conclusion, we provide a comprehensive comparison of the molecular properties and relationship of the five mammalian isoforms ATP13A1-5. Based on a phylogenetic analysis and supported by a distinct subcellular targeting, topology and functional complementation potential, we provide strong support for the division of the animal P5-type ATPases into a P5A and P5B group. The P5A most likely carries out a general cellular function in the ER related to protein folding and quality control, whereas P5B ATPases have evolved cell-specific functions in the late endo-/lysosomal network. Evolution of animals has been accompanied not only by development of a central nervous system, but also by the appearance of specialized epithelial glandular cells that are essential for diverse functions such as slime production in lungs to milk secretion, which strongly depend on an endo-/lysosomal system to carry out extensive exo- and endocytosis. Further studies will have to reveal the specific roles of P5B ATPases in shaping evolution and development of animals.

## Supporting information

S1 Fig Overview of P5B gene duplication events in animal evolution. The phylogenetic tree depicts (1) Gene duplication in deuterostomia evolution of the P5B_ANCESTOR_ orthologue resulted in an ATP13A2 and ATP13A3 isoform. A single P5B_INV_ isoform (invertebrates) is present in most protostomia species. (2) Gene duplication of ATP13A3 into an ATP13A3 and ATP13A4 isoform. (3) Gene duplication of ATP13A4 into an ATP13A4 and ATP13A5 isoform.
(PDF)

S2 Fig Tissue distribution of P5-type ATPase mRNA expression. Analysis of publically available mRNA expression data shows a P5-type ATPase expression profile that is tissue and development specific in human and mouse. **A.** Comparing expression of ATP13A1-5 mRNA in human tissue samples shows a general presence of ATP13A1 and ATP13A3 mRNA across all tissues with lower expression in muscoskeletal muscle (ATP13A1 and ATP13A3) and nervous and reproductive systems (ATP13A3). ATP13A2 strongly expresses in the nervous system, whereas ATP13A4 is mainly expressed in the respiratory systems (lungs, pharynx, trachea) and integumentary system (skin and epidermis) while ATP13A5 strongly express in the sensory organs (eye and nose) and integumentary systems. Number of samples included for each tissue is included on the right. **B.** During mouse development, ATP13A3 appears to be strongly expressed at the early prenatal period (2–4 days of development), while ATP13A4 and ATP13A5 mRNA shows expression in the post-natal period. Only developmental data for ATP13A3-5 are depicted, because only these isoforms show differential expression during development. **C-D.** Expression profiles of ATP13A4 and ATP13A5 mRNA in human cell and tissue samples. Only the tissues with the highest expression are shown. Both genes express highly in cuboidal alveolar type 2 cells (black arrow) and in tissues of the sexual organs (light grey arrows: ATP13A4 epididymal corpus in testis, ATP13A5 mammary gland of the breast). ATP13A4 express highly in the oral mucosa and gingiva of the mouth (light blue arrows). ATP13A5 shows a general high expression in epidermis and dermis cells but also shows specific expression in the nasal epithelium and cells of nasal organotypic tissue cultures (dark blue arrows). ATP13A1-3 show a much broader tissue distribution and are not depicted.
(PDF)

S3 Fig Purification of yeast Ypk9p (WT and D781N). **A-B.** The N-terminal 10xHis-tagged versions of Ypk9p (**A**) and Ypk9 (D781N) (**B**) were purified to relative homogeneity as described in materials and methods by utilizing Ni^2+^ affinity chromatography. The resulting proteins were found at the expected size of ~170 kDa at high purity. 15 μl of sample for each fraction during purification was separated using SDS-PAGE and visualized with Coomassie blue staining. Flowthrough (FT), wash (W1-4), elution (E1-4) and marker (Mk) is indicated.
(PDF)

S4 Fig Characterization of the affinity-purified ATP13A1 antibody for immunoblotting. **A.** Topology model of ATP13A1 indicating the peptide recognition sequences of the ATP13A1 SY2459 (AA 543–557 in hATP13A1) **B-C.** Microsomal fractions of COS-8 cells transiently transfected with ATP13A1 or ATP13A1 fusion proteins (N-terminal GFP-tag) were applied. As a negative control, the microsomal fraction of non-transfected COS-8 cells was loaded. Blots were incubated with ATP13A1 SY2459 antibody, which recognizes full-length mATP13A1 (133 kDa). In (**C**) blots were incubated with the antibody in absence or presence of excess quantities of the immunizing peptide.
(PDF)

S5 Fig Catalytic dead mutants display similar targeting as WT proteins. **A.** The catalytically inactive ATP13A1 mutant D530N is targeted to the ER, overlapping with the ER marker SERCA2b. **B.** Like WT ATP13A2, the catalytically inactive mutant hATP13A2-D508N reaches the late endosomal compartment (visualized with GFP-labeled RAB7). Scale bar represents 20 μm.
(PDF)

S6 Fig ATP13A1 contains an additional N-terminal hairpin. **A.** Predicted topology model of ATP13A1 with 12 TM helices (M) (TMHMM v2). On top of the figure, the numbering of membrane helices is indicated: M1 corresponds to the first M helix that is present in all P-type ATPases, whereas more upstream helices are referred to as Ma and Mb, with Mb the most N-terminal helix. **B-L.** Fluorescence protease protection assay. In HeLa cells, WT ATP13A1 with N- (**D**) or C-terminal (**E**) GFP-tag, Mb-Ma-C-GFP (residues 1–197) (**I**), Mb-C-GFP (residues 1–95) (**J**) or controls (ERO-GFP (**C**) or GFP (**B**) were transiently transfected and subjected to FPP. Pictures at 0’, 3’ and 5’ are depicted. Numbers in circles correspond to positions indicated in the cartoons F-H. + and–in circles indicate positive or negative control. **F.** Experimentally verified topology model of ATP13A1 depicting 12 TM helices. **G-H.** Cartoon depicting the Ma and Mb-Ma constructs (in the cartoon, a C-terminal GFP-tag is indicated). **K-L.** HeLa cells were transiently co-transfected with Mb (C-terminal mCherry-tag) or Mb-Ma (C-terminal GFP-tag) and different cellular markers. Mb co-localizes with Rab7, a marker for late endo-/lysosomes (**K**), whereas Mb-Ma co-localizes with SERCA2b, an ER resident protein. Scale bar represents 40 μm (B-J) or 20 μm (K-L).
(PDF)

S7 Fig The P5B ATPases ATP13A3 and ATP13A4 share topology with ATP13A2. **A.** Topology model of ATP13A2 comprising 10 TM helices and one membrane-associated helix, not spanning the membrane. **B-E.** Fluorescence protease protection (FPP) assay. WT ATP13A3 (N- (**B**) or C-terminal (**C**) GFP-tag) or WT ATP13A4 (N- (**D**) or C-terminal (**E**) GFP-tag) were transiently transfected in HeLa cells and subjected to FPP. Pictures were acquired at 0’, 3’ and 5’. Numbers in circles correspond to the position of the GFP-tag in (A). Scale bar represents 40 μm.
(PDF)

S8 Fig Complementation assay with GFP-labeled ATP13A1 constructs. Both the N- and C-terminal GFP labeled ATP13A1 constructs provide a functional complementation of the *spf1*^*-*^ deletion phenotype in yeast. This is not seen with a catalytic dead mutant of ATP13A1 (D530N). This proves that the N- and C-terminal fusion constructs remain functionally active and are not affected by the tag.
(PDF)

S1 Table Accession numbers of sequences included in the phylogenetic analysis. Sequence information of the P5 isoforms included in the phylogenetic analysis of Figs 1 and 2. n.s. not specified; A1, P5A isoform; B1-5, number of P5B isoforms per species; P5BINV, P5B invertebrates.
(PDF)
