# Supplementary figures and images for: Parkinson disease related ATP13A2 evolved early in animal evolution
Source: PLoS One. 2018 Mar 5;13(3):e0193228. doi: 10.1371/journal.pone.0193228 (PMC5837089; doi:10.1371/journal.pone.0193228)

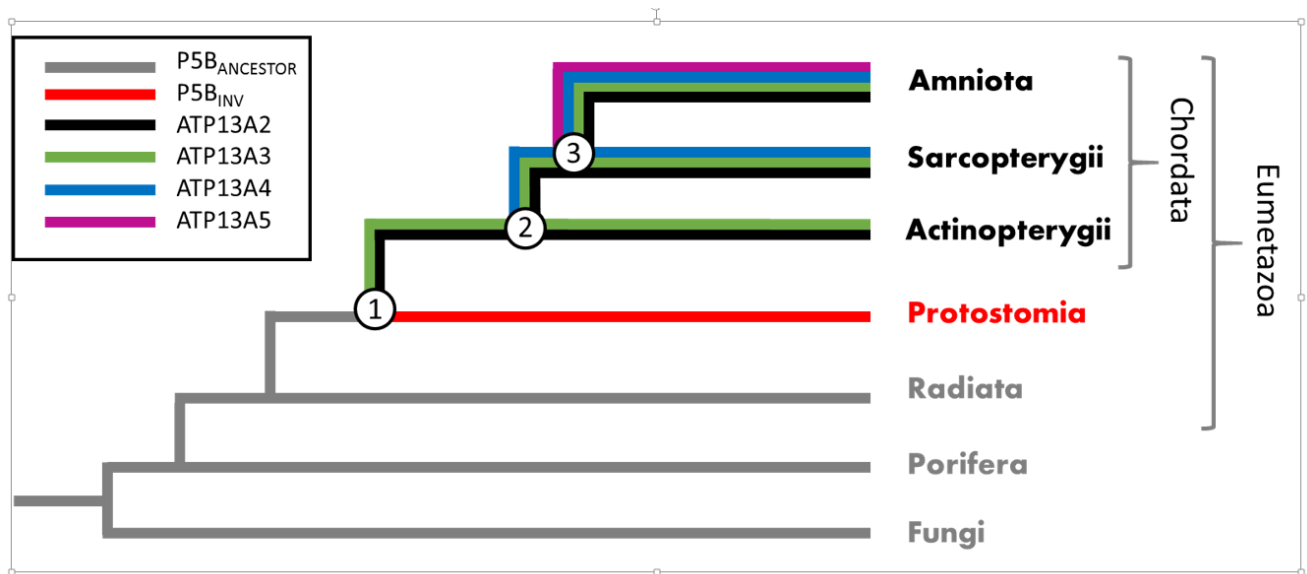

Suppl. Fig. 1. Overview of P5B gene duplication events in animal evolution

Supplement: S1 Fig — The phylogenetic tree depicts (1) Gene duplication in deuterostomia evolution of the P5BANCESTOR orthologue resulted in an ATP13A2 and ATP13A3 isoform. A single P5BINV isoform (invertebrates) is present in most protostomia species. (2) Gene duplication of ATP13A3 into an ATP13A3 and ATP13A4 isoform. (3) Gene duplication of ATP13A4 into an ATP13A4 and ATP13A5 isoform. (PDF) [file pone.0193228.s001.pdf]

Suppl. Fig. 2

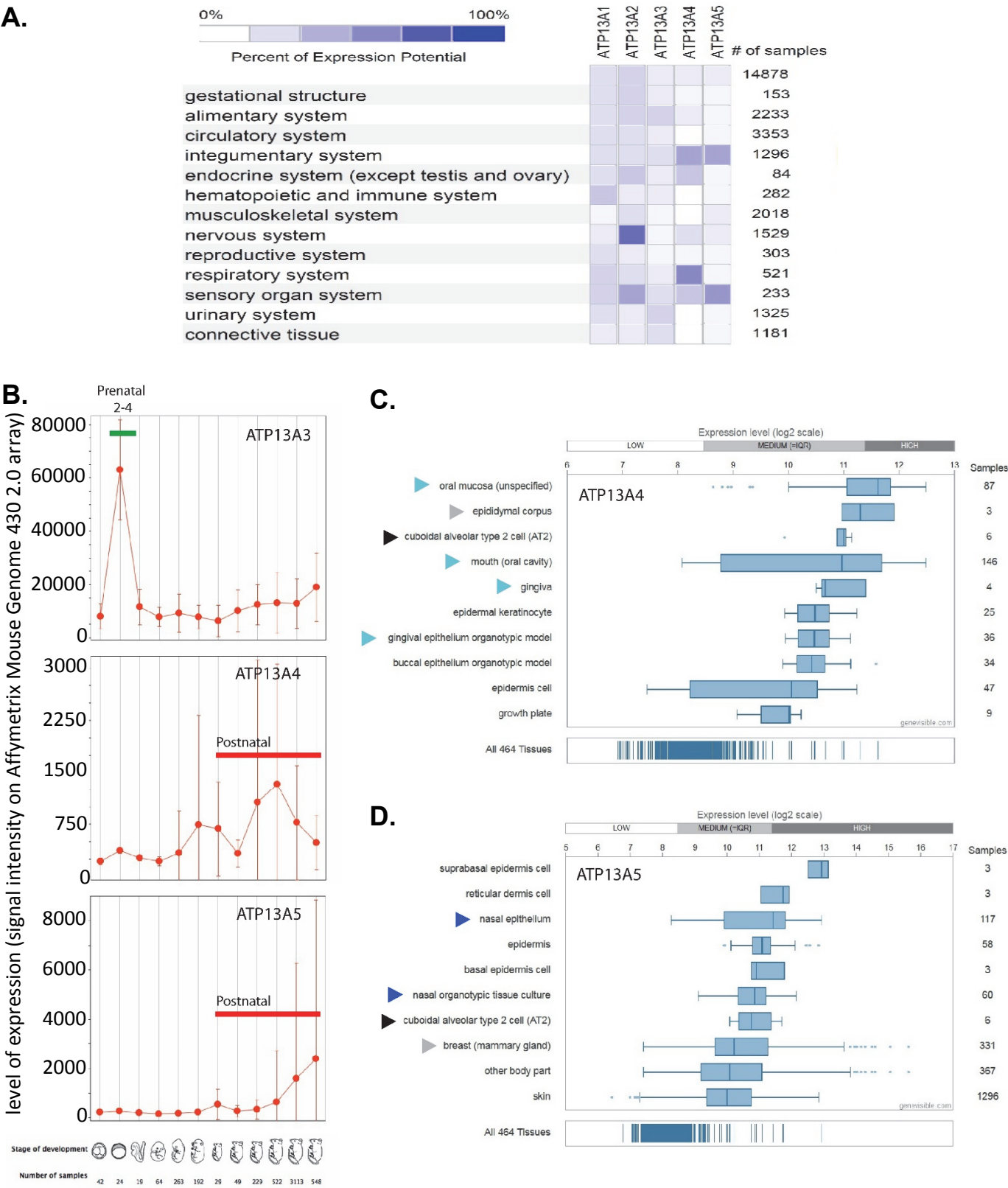

Suppl. Fig. 2. Tissue distribution of P5-type ATPase mRNA expression

Supplement: S2 Fig — Analysis of publically available mRNA expression data shows a P5-type ATPase expression profile that is tissue and development specific in human and mouse. A. Comparing expression of ATP13A1-5 mRNA in human tissue samples shows a general presence of ATP13A1 and ATP13A3 mRNA across all tissues with lower expression in muscoskeletal muscle (ATP13A1 and ATP13A3) and nervous and reproductive systems (ATP13A3). ATP13A2 strongly expresses in the nervous system, whereas ATP13A4 is mainly expressed in the respiratory systems (lungs, pharynx, trachea) and integumentary system (skin and epidermis) while ATP13A5 strongly express in the sensory organs (eye and nose) and integumentary systems. Number of samples included for each tissue is included on the right. B. During mouse development, ATP13A3 appears to be strongly expressed at the early prenatal period (2–4 days of development), while ATP13A4 and ATP13A5 mRNA shows expression in the post-natal period. Only developmental data for ATP13A3-5 are depicted, because only these isoforms show differential expression during development. C-D. Expression profiles of ATP13A4 and ATP13A5 mRNA in human cell and tissue samples. Only the tissues with the highest expression are shown. Both genes express highly in cuboidal alveolar type 2 cells (black arrow) and in tissues of the sexual organs (light grey arrows: ATP13A4 epididymal corpus in testis, ATP13A5 mammary gland of the breast). ATP13A4 express highly in the oral mucosa and gingiva of the mouth (light blue arrows). ATP13A5 shows a general high expression in epidermis and dermis cells but also shows specific expression in the nasal epithelium and cells of nasal organotypic tissue cultures (dark blue arrows). ATP13A1-3 show a much broader tissue distribution and are not depicted. (PDF) [file pone.0193228.s002.pdf]

## Suppl. Fig. 3

**A.**

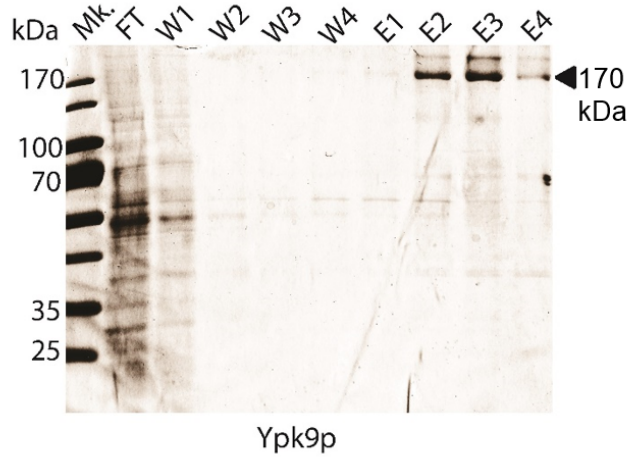

**B.**

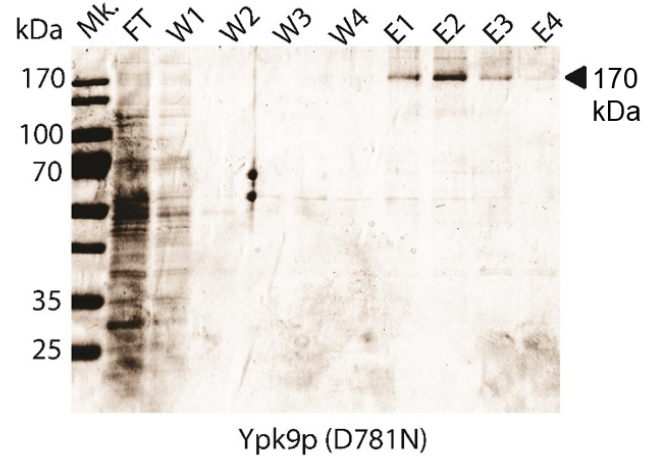

**Suppl. Fig. 3. Purification of yeast Ypk9p and Ypk9p (D781N)**

Supplement: S3 Fig — A-B. The N-terminal 10xHis-tagged versions of Ypk9p (A) and Ypk9 (D781N) (B) were purified to relative homogeneity as described in materials and methods by utilizing Ni2+ affinity chromatography. The resulting proteins were found at the expected size of ~170 kDa at high purity. 15 μl of sample for each fraction during purification was separated using SDS-PAGE and visualized with Coomassie blue staining. Flowthrough (FT), wash (W1-4), elution (E1-4) and marker (Mk) is indicated. (PDF) [file pone.0193228.s003.pdf]

## Suppl. Fig. 5

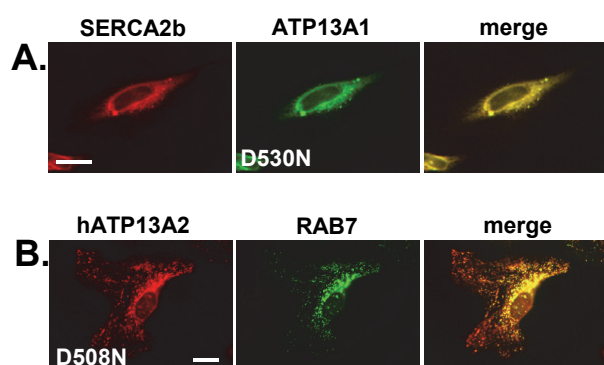

Suppl. Fig. 5. Catalytic dead mutants display similar targeting as WT proteins

Supplement: S5 Fig — A. The catalytically inactive ATP13A1 mutant D530N is targeted to the ER, overlapping with the ER marker SERCA2b. B. Like WT ATP13A2, the catalytically inactive mutant hATP13A2-D508N reaches the late endosomal compartment (visualized with GFP-labeled RAB7). Scale bar represents 20 μm. (PDF) [file pone.0193228.s005.pdf]

# Suppl. Fig. 6

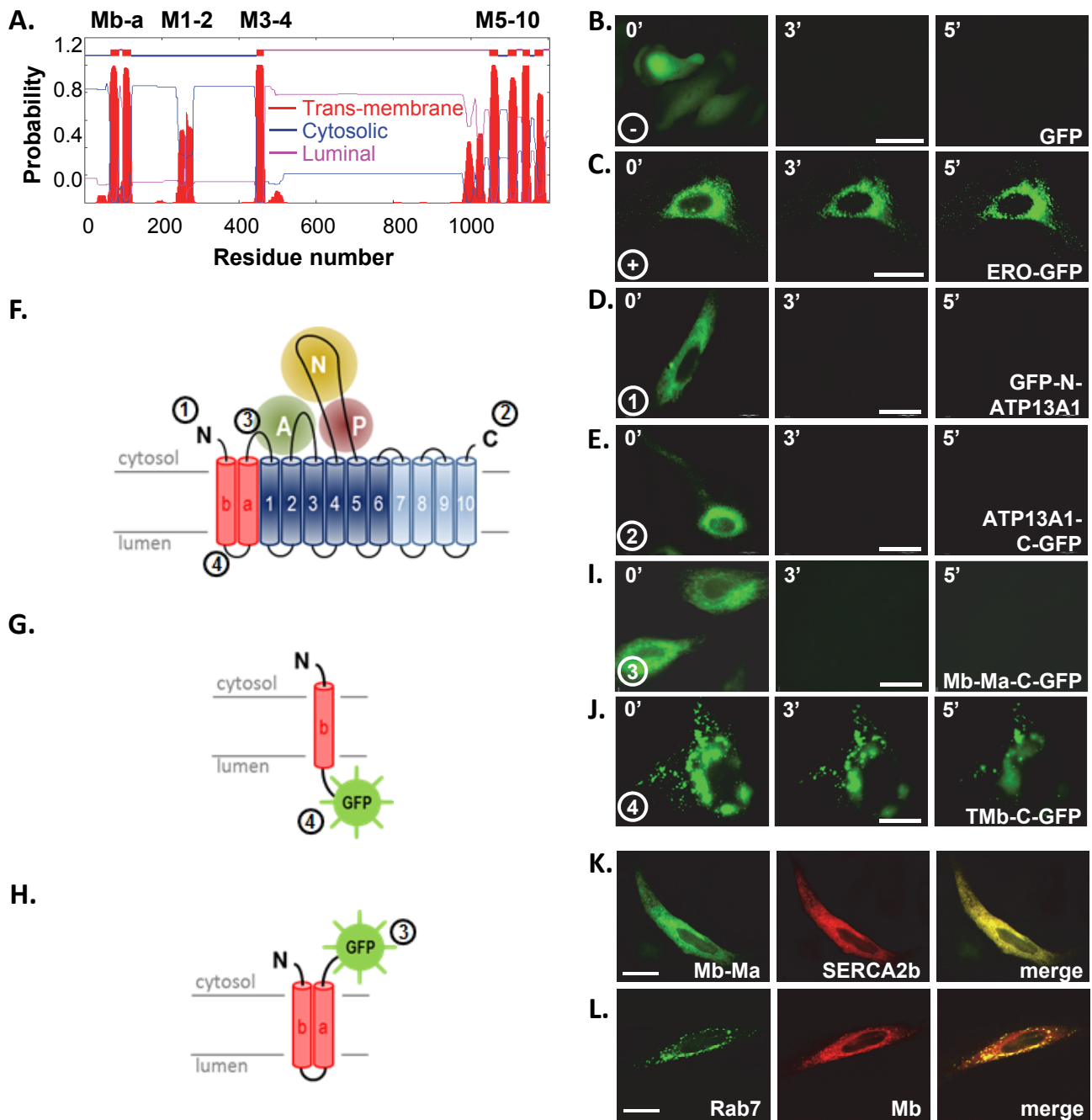

Suppl. Fig. 6. ATP13A1 contains an additional N-terminal hairpin.

Supplement: S6 Fig — A. Predicted topology model of ATP13A1 with 12 TM helices (M) (TMHMM v2). On top of the figure, the numbering of membrane helices is indicated: M1 corresponds to the first M helix that is present in all P-type ATPases, whereas more upstream helices are referred to as Ma and Mb, with Mb the most N-terminal helix. B-L. Fluorescence protease protection assay. In HeLa cells, WT ATP13A1 with N- (D) or C-terminal (E) GFP-tag, Mb-Ma-C-GFP (residues 1–197) (I), Mb-C-GFP (residues 1–95) (J) or controls (ERO-GFP (C) or GFP (B) were transiently transfected and subjected to FPP. Pictures at 0’, 3’ and 5’ are depicted. Numbers in circles correspond to positions indicated in the cartoons F-H. + and–in circles indicate positive or negative control. F. Experimentally verified topology model of ATP13A1 depicting 12 TM helices. G-H. Cartoon depicting the Ma and Mb-Ma constructs (in the cartoon, a C-terminal GFP-tag is indicated). K-L. HeLa cells were transiently co-transfected with Mb (C-terminal mCherry-tag) or Mb-Ma (C-terminal GFP-tag) and different cellular markers. Mb co-localizes with Rab7, a marker for late endo-/lysosomes (K), whereas Mb-Ma co-localizes with SERCA2b, an ER resident protein. Scale bar represents 40 μm (B-J) or 20 μm (K-L). (PDF) [file pone.0193228.s006.pdf]

# Suppl. Fig. 7

A.

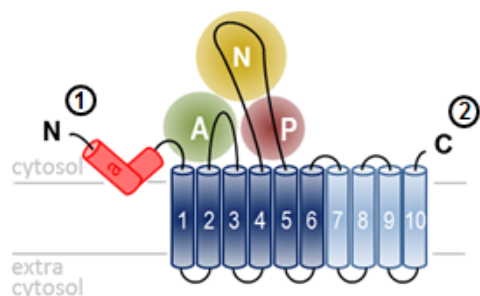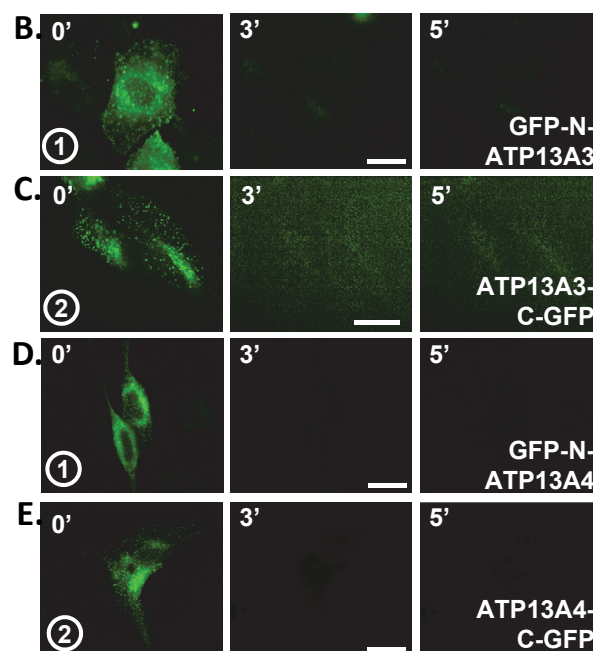

Suppl. Fig. 7. P5B ATPases ATP13A3 and ATP13A4 share topology with ATP13A2

Supplement: S7 Fig — A. Topology model of ATP13A2 comprising 10 TM helices and one membrane-associated helix, not spanning the membrane. B-E. Fluorescence protease protection (FPP) assay. WT ATP13A3 (N- (B) or C-terminal (C) GFP-tag) or WT ATP13A4 (N- (D) or C-terminal (E) GFP-tag) were transiently transfected in HeLa cells and subjected to FPP. Pictures were acquired at 0’, 3’ and 5’. Numbers in circles correspond to the position of the GFP-tag in (A). Scale bar represents 40 μm. (PDF) [file pone.0193228.s007.pdf]

# Suppl. Fig. 8

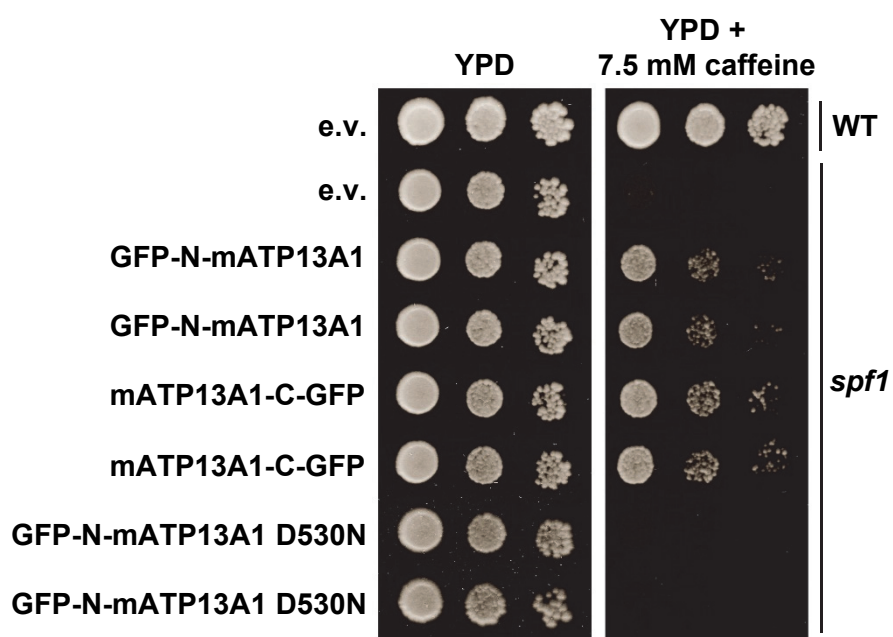

Suppl. Fig. 8. Complementation assay with GFP-labeled ATP13A1 constructs

Supplement: S8 Fig — Both the N- and C-terminal GFP labeled ATP13A1 constructs provide a functional complementation of the spf1- deletion phenotype in yeast. This is not seen with a catalytic dead mutant of ATP13A1 (D530N). This proves that the N- and C-terminal fusion constructs remain functionally active and are not affected by the tag. (PDF) [file pone.0193228.s008.pdf]
