# Supplementary material for: Parkinson disease related ATP13A2 evolved early in animal evolution
Source: PLoS One. 2018 Mar 5;13(3):e0193228. doi: 10.1371/journal.pone.0193228 (PMC5837089; doi:10.1371/journal.pone.0193228)
Supplement: S4 Fig — A. Topology model of ATP13A1 indicating the peptide recognition sequences of the ATP13A1 SY2459 (AA 543–557 in hATP13A1) B-C. Microsomal fractions of COS-8 cells transiently transfected with ATP13A1 or ATP13A1 fusion proteins (N-terminal GFP-tag) were applied. As a negative control, the microsomal fraction of non-transfected COS-8 cells was loaded. Blots were incubated with ATP13A1 SY2459 antibody, which recognizes full-length mATP13A1 (133 kDa). In (C) blots were incubated with the antibody in absence or presence of excess quantities of the immunizing peptide. (PDF) [file pone.0193228.s004.pdf]

Suppl. Fig. 4

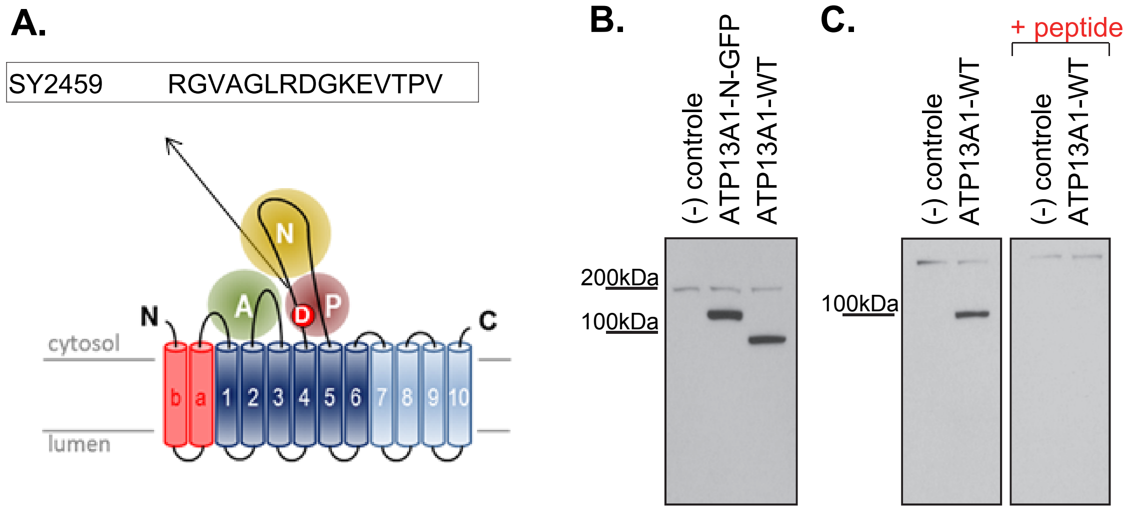

Suppl. Fig. 4. Characterization of the affinity-purified ATP13A1 antibody for immunoblotting.
