# Supplementary material for: Parkinson disease related ATP13A2 evolved early in animal evolution
Source: PLoS One. 2018 Mar 5;13(3):e0193228. doi: 10.1371/journal.pone.0193228 (PMC5837089; doi:10.1371/journal.pone.0193228)
Supplement: S1 Table — Sequence information of the P5 isoforms included in the phylogenetic analysis of Figs 1 and 2. n.s. not specified; A1, P5A isoform; B1-5, number of P5B isoforms per species; P5BINV, P5B invertebrates. (PDF) [file pone.0193228.s009.pdf]

Protein name	Species	Subgroup	Database	Reference	P5 group	Notes
ATP13A1	*Homo sapiens*	*Chordata Mammalia*	Uniprot	Q9HD20	ATP13A1	
ATP13A2	*Homo sapiens*	*Chordata Mammalia*	Uniprot	Q9NQ11	ATP13A2	
ATP13A3	*Homo sapiens*	*Chordata Mammalia*	Uniprot	Q9H7F0	ATP13A3	
ATP14A4	*Homo sapiens*	*Chordata Mammalia*	Uniprot	Q4VNC1	ATP13A4	
ATP13A5	*Homo sapiens*	*Chordata Mammalia*	Uniprot	Q4VNC0	ATP13A5	
Trichinella spiralis A1	*Trichinella spiralis*	*Nematoda Enoplea*	NCBI	KRY39294.1	ATP13A1	Lacks start Methionine
Trichinella spiralis B1	*Trichinella spiralis*	*Nematoda Enoplea*	NCBI	KRY41564.1	P5BINV	
Mnemiopsis leidyi A1	*Mnemiopsis leidyi*	*Ctenophora Tentaculata*	NIH	ML078814a	ATP13A1	
Mnemiopsis leidyi B1	*Mnemiopsis leidyi*	*Ctenophora Tentaculata*	NIH	ML03044a	n.s.	
Trichoplax adhaerens A1	*Trichoplax adhaerens*	*Parazoa Placozoa*	NCBI	XP_002109418.1	ATP13A1	
Trichoplax adhaerens B3	*Trichoplax adhaerens*	*Parazoa Placozoa*	NCBI	XP_002113328.1	n.s.	Lacking part of N-terminal sequence
Trichoplax adhaerens B2	*Trichoplax adhaerens*	*Parazoa Placozoa*	NCBI	XP_002117690.1	n.s.	Lacking part of N-terminal sequence + other gaps
Trichoplax adhaerens B1	*Trichoplax adhaerens*	*Parazoa Placozoa*	NCBI	XP_002117689.1	n.s.	Lacking part of N-terminal sequence + other gaps
Exaiptasia pallida A1	*Exaiptasia pallida*	*Cnidaria Anthozoa*	NCBI	KXJ19419.1	ATP13A1	
Exaiptasia pallida B2	*Exaiptasia pallida*	*Cnidaria Anthozoa*	NCBI	KXJ16319.1	n.s.	A 'J' in the motif 'FLJ' has been changed to an 'X'
Amphimedon queenslandica A1	*Amphimedon queenslandica*	*Porifera Demospongiae*	NCBI	XP_011403965.1	ATP13A1	
Amphimedon queenslandica B1	*Amphimedon queenslandica*	*Porifera Demospongiae*	NCBI	XP_011405042.1	n.s.	
Athalia rosae A1	*Athalia rosae*	*Arthropoda Insecta*	NCBI	XP_012262729.1	ATP13A1	
Athalia rosae B2	*Athalia rosae*	*Arthropoda Insecta*	NCBI	XP_012263211.1	P5BINV	
Athalia rosae B1	*Athalia rosae*	*Arthropoda Insecta*	NCBI	XP_012255739.1	P5BINV	
Atta cephalotes A1	*Atta cephalotes*	*Arthropoda Insecta*	NCBI	XP_012055862.1	ATP13A1	
Atta cephalotes B1	*Atta cephalotes*	*Arthropoda Insecta*	NCBI	XP_012060096.1	P5BINV	
Nasonia vitripennis A1	*Nasonia vitripennis*	*Arthropoda Insecta*	NCBI	NP_001135438.1	ATP13A1	
Nasonia vitripennis B3	*Nasonia vitripennis*	*Arthropoda Insecta*	NCBI	XP_008206785.1	P5BINV	
Nasonia vitripennis B2	*Nasonia vitripennis*	*Arthropoda Insecta*	NCBI	XP_016842428.1	P5BINV	

Nasonia vitripennis B1	*Nasonia vitripennis*	*Arthropoda Insecta*	NCBI	XP_008204138.1	P5BINV	
Danaus plexippus A1	*Danaus plexippus*	*Arthropoda Insecta*	KEGG	dpl:KGM_02473	ATP13A1	
Danaus plexippus B1	*Danaus plexippus*	*Arthropoda Insecta*	KEGG	dpl:KGM_03862	ATP13A2	
Danaus plexippus B2	*Danaus plexippus*	*Arthropoda Insecta*	KEGG	dpl:KGM_13021	P5BINV	
Apis mellifera A1	*Apis mellifera*	*Arthropoda Insecta*	NCBI	XP_396194.3	ATP13A1	
Apis mellifera B1	*Apis mellifera*	*Arthropoda Insecta*	NCBI	XP_016771871.1	P5BINV	
Apis mellifera B2	*Apis mellifera*	*Arthropoda Insecta*	NCBI	XP_394006.4	P5BINV	
Necator americanus A1	*Necator americanus*	*Nematoda Secernentea*	NCBI	ETN69903.1	ATP13A1	
Necator americanus B1	*Necator americanus*	*Nematoda Secernentea*	NCBI	ETN76092.1	P5BINV	
Caenorhabditis elegans A1	*Caenorhabditis elegans*	*Nematoda Chromodorea*	NCBI	CAB05683.2	ATP13A1	
Caenorhabditis elegans B1	*Caenorhabditis elegans*	*Nematoda Chromodorea*	NCBI	CCD71142.1	P5BINV	
Caenorhabditis elegans B3	*Caenorhabditis elegans*	*Nematoda Chromodorea*	NCBI	CAA94236.2	P5BINV	
Caenorhabditis elegans B2	*Caenorhabditis elegans*	*Nematoda Chromodorea*	NCBI	CCD62518.1	P5BINV	
Biomphalaria glabrata A1	*Biomphalaria glabrata*	*Mollusca Gastropoda*	NCBI	XP_013085670.1	ATP13A1	
Biomphalaria glabrata B1	*Biomphalaria glabrata*	*Mollusca Gastropoda*	NCBI	XP_013085041.1	P5BINV	
Aplysia californica A1	*Aplysia californica*	*Mollusca Gastropoda*	NCBI	XP_005097645.1	ATP13A1	
Aplysia californica B1	*Aplysia californica*	*Mollusca Gastropoda*	NCBI	XP_012945191.1	ATP13A2	
Crassostrea gigas A1	*Crassostrea gigas*	*Mollusca Bivalvia*	NCBI	XP_011452247.1	ATP13A1	
Crassostrea gigas B1	*Crassostrea gigas*	*Mollusca Bivalvia*	NCBI	XP_011444154.1	ATP13A2	
Crassostrea gigas B2	*Crassostrea gigas*	*Mollusca Bivalvia*	NCBI	XP_011413198.1	P5BINV	
Crassostrea gigas B3	*Crassostrea gigas*	*Mollusca Bivalvia*	NCBI	EKC40831.1	P5BINV	
Strongylocentrotus purpuratus A1	*Strongylocentrotus purpuratus*	*Echinodermata Echinoidea*	EchinoBase	SPU_020368.1	ATP13A1	
Strongylocentrotus purpuratus B1	*Strongylocentrotus purpuratus*	*Echinodermata Echinoidea*	NCBI	XP_011676514.1	-	
Strongylocentrotus purpuratus B4	*Strongylocentrotus purpuratus*	*Echinodermata Echinoidea*	NCBI	XP_011661493.1	-	
Strongylocentrotus purpuratus B2	*Strongylocentrotus purpuratus*	*Echinodermata Echinoidea*	NCBI	XP_011660791.1	-	SP instead of PP in TM4
Strongylocentrotus purpuratus B3	*Strongylocentrotus purpuratus*	*Echinodermata Echinoidea*	EchinoBase	SPU_002465.3a	-	
Drosophila melanogaster A1	*Drosophila melanogaster*	*Arthropoda Insecta*	NCBI	NP_609490.1	ATP13A1	
Drosophila melanogaster B1	*Drosophila melanogaster*	*Arthropoda Insecta*	NCBI	NP_001096849.1	P5BINV	

Anopheles gambiae A1	*Anopheles gambiae*	*Arthropoda Insecta*	NCBI	XP_317372.3	ATP13A1	
Anopheles gambiae B1	*Anopheles gambiae*	*Arthropoda Insecta*	NCBI	XP_309375.4	P5BINV	Lacks start methionine
Branchiostoma floridae A1	*Branchiostoma floridae*	*Chordata Cephalochordat a*	NCBI	XP_002592452.1*	ATP13A1	* Gaps in sequence filled (see table text)
Ciona intestinalis A1	*Ciona intestinalis*	*Chordata Tunicata*	NCBI	XP_009859591.1	ATP13A1	
Ciona intestinalis B1	*Ciona intestinalis*	*Chordata Tunicata*	NCBI	XP_009861572.1	ATP13A3	
Sarcophilus harrisii A1	*Sarcophilus harrisii*	*Chordata Mammalia*	KEGG	shr:100914699	ATP13A1	
Sarcophilus harrisii B2	*Sarcophilus harrisii*	*Chordata Mammalia*	KEGG	shr:100933481	ATP13A3	
Sarcophilus harrisii B3	*Sarcophilus harrisii*	*Chordata Mammalia*	KEGG	shr:100930518	ATP13A4	
Sarcophilus harrisii B1	*Sarcophilus harrisii*	*Chordata Mammalia*	KEGG	shr:100924559	ATP13A2	
Pan troglodytes B1	*Pan troglodytes*	*Chordata Mammalia*	KEGG	ptr:456525	ATP13A2	
Pan troglodytes B2	*Pan troglodytes*	*Chordata Mammalia*	KEGG	ptr:471045	ATP13A3	
Pan troglodytes B3	*Pan troglodytes*	*Chordata Mammalia*	KEGG	ptr:460940	ATP13A4	
Pan troglodytes B4	*Pan troglodytes*	*Chordata Mammalia*	KEGG	ptr:460939	ATP13A5	
Bos Taurus A1	*Bos taurus*	*Chordata Mammalia*	KEGG	bta:534369	ATP13A1	
Bos Taurus B1	*Bos taurus*	*Chordata Mammalia*	KEGG	bta:510792	ATP13A2	
Bos Taurus B2	*Bos taurus*	*Chordata Mammalia*	KEGG	bta:523889	ATP13A3	
Bos Taurus B3	*Bos taurus*	*Chordata Mammalia*	KEGG	bta:521728	ATP13A4	
Bos Taurus B4	*Bos taurus*	*Chordata Mammalia*	KEGG	bta:509596	ATP13A5	
Mus musculus A1	*Mus musculus*	*Chordata Mammalia*	NCBI	NP_573487.2	ATP13A1	
Mus musculus B1	*Mus musculus*	*Chordata Mammalia*	NCBI	AAH42661.1	ATP13A2	
Mus musculus B2	*Mus musculus*	*Chordata Mammalia*	KEGG	mmu:224088	ATP13A3	
Mus musculus B3	*Mus musculus*	*Chordata Mammalia*	Uniprot	Q5XF90.1	ATP13A4	
Mus musculus B4	*Mus musculus*	*Chordata Mammalia*	NCBI	NP_783581.2	ATP13A5	
Rattus norvegicus A1	*Rattus norvegicus*	*Chordata Mammalia*	KEGG	rno:290673	ATP13A1	
Rattus norvegicus B4	*Rattus norvegicus*	*Chordata Mammalia*	KEGG	rno:303856	ATP13A5	
Rattus norvegicus B1	*Rattus norvegicus*	*Chordata Mammalia*	KEGG	rno:362645	ATP13A2	
Rattus norvegicus B3	*Rattus norvegicus*	*Chordata Mammalia*	KEGG	rno:288026	ATP13A4	
Rattus norvegicus B2	*Rattus norvegicus*	*Chordata Mammalia*	NCBI	EDL78157.1	ATP13A3	

Gallus gallus A1	*Gallus gallus*	*Chordata Aves*	NCBI	XP_004950884.1	ATP13A1	
Gallus gallus B1	*Gallus gallus*	*Chordata Aves*	KEGG	gga:424898	ATP13A3	
Gallus gallus B2	*Gallus gallus*	*Chordata Aves*	NCBI	NP_001026485.1	ATP13A4	
Gallus gallus B3	*Gallus gallus*	*Chordata Aves*	NCBI	XP_422713.3	ATP13A5	
Danio rerio A1	*Danio rerio*	*Chordata Actinopterygii*	KEGG	dre:334466	ATP13A1	
Danio rerio B1	*Danio rerio*	*Chordata Actinopterygii*	KEGG	dre:568666	ATP13A2	
Danio rerio B2	*Danio rerio*	*Chordata Actinopterygii*	KEGG	dre:572029	ATP13A3	
Equus callabus A1	*Equus callabus*	*Chordata Mammalia*	KEGG	ecb:100071205	ATP13A1	
Equus callabus B2	*Equus callabus*	*Chordata Mammalia*	KEGG	ecb:100069072	ATP13A3	
Equus callabus B4	*Equus callabus*	*Chordata Mammalia*	KEGG	ecb:100068956	ATP13A5	
Equus callabus B1	*Equus callabus*	*Chordata Mammalia*	KEGG	ecb:100053155	ATP13A2	
Equus callabus B3	*Equus callabus*	*Chordata Mammalia*	KEGG	ecb:100068981	ATP13A4	
Anolis carolinensis A1	*Anolis carolinensis*	*Chordata Reptilia*	KEGG	acs:100557108	ATP13A1	
Anolis carolinensis B4	*Anolis carolinensis*	*Chordata Reptilia*	KEGG	acs:100557913	ATP13A5	
Anolis carolinensis B3	*Anolis carolinensis*	*Chordata Reptilia*	KEGG	acs:100555953	ATP13A3	
Anolis carolinensis B2	*Anolis carolinensis*	*Chordata Reptilia*	KEGG	acs:100561002	ATP13A3	
Sus scrofa A1	*Sus scrofa*	*Chordata Mammalia*	KEGG	ssc:100738340	ATP13A1	
Sus scrofa B1	*Sus scrofa*	*Chordata Mammalia*	KEGG	ssc:102165725	ATP13A2	
Sus scrofa B2	*Sus scrofa*	*Chordata Mammalia*	KEGG	ssc:100522900	ATP13A3	
Sus scrofa B3	*Sus scrofa*	*Chordata Mammalia*	KEGG	ssc:100157346	ATP13A4	
Sus scrofa B4	*Sus scrofa*	*Chordata Mammalia*	KEGG	ssc:100154528	ATP13A5	
Python bivittatus A1	*Python bivittatus*	*Chordata Reptilia*	NCBI	XP_007421473.1	ATP13A1	
Python bivittatus B1	*Python bivittatus*	*Chordata Reptilia*	NCBI	XP_007440981.1	ATP13A2	
Python bivittatus B3	*Python bivittatus*	*Chordata Reptilia*	NCBI	XP_007442874.1	ATP13A3	
Python bivittatus B2	*Python bivittatus*	*Chordata Reptilia*	NCBI	XP_007442657.1	ATP13A3	
Python bivittatus B4	*Python bivittatus*	*Chordata Reptilia*	NCBI	XP_007433005.1	ATP13A4	
Falco peregrinus A1	*Falco peregrinus*	*Chordata Aves*	KEGG	fpg:101910338	ATP13A1	
Falco peregrinus B1	*Falco peregrinus*	*Chordata Aves*	KEGG	fpg:101918322	ATP13A2	
Falco peregrinus B2	*Falco peregrinus*	*Chordata Aves*	KEGG	fpg:101916026	ATP13A3	
Falco peregrinus B4	*Falco peregrinus*	*Chordata Aves*	KEGG	fpg:101913032	ATP13A5	

Falco peregrinus B3	*Falco peregrinus*	*Chordata Aves*	KEGG	fpg:101916365	ATP13A4	
Anas platyrhynchos A1	*Anas platyrhynchos*	*Chordata Aves*	KEGG	apla:101804697	ATP13A1	
Anas platyrhynchos B1	*Anas platyrhynchos*	*Chordata Aves*	KEGG	apla:101793641	ATP13A2	
Anas platyrhynchos B2	*Anas platyrhynchos*	*Chordata Aves*	KEGG	apla:101794853	ATP13A3	
Anas platyrhynchos B4	*Anas platyrhynchos*	*Chordata Aves*	KEGG	apla:101802354	ATP13A5	
Anas platyrhynchos B3	*Anas platyrhynchos*	*Chordata Aves*	KEGG	apla:101794117	ATP13A4	
Pelodiscus sinensis A1	*Pelodiscus sinensis*	*Chordata Reptilia*	KEGG	pss:102446500	ATP13A1	
Pelodiscus sinensis B1	*Pelodiscus sinensis*	*Chordata Reptilia*	KEGG	pss:102446024	ATP13A2	
Pelodiscus sinensis B2	*Pelodiscus sinensis*	*Chordata Reptilia*	KEGG	pss:102451280	ATP13A3	
Pelodiscus sinensis B3	*Pelodiscus sinensis*	*Chordata Reptilia*	KEGG	pss:102456702	ATP13A5	
Maylandia zebra A1	*Maylandia zebra*	*Chordata Actinopterygii*	KEGG	mze:101481768	ATP13A1	
Maylandia zebra B1	*Maylandia zebra*	*Chordata Actinopterygii*	KEGG	mze:101478591	ATP13A2	
Maylandia zebra B2	*Maylandia zebra*	*Chordata Actinopterygii*	KEGG	mze:101474717	ATP13A3	
Salmo salar A1	*Salmo salar*	*Chordata Actinopterygii*	NCBI	XP_014059831.1	ATP13A1	
Salmo salar A2	*Salmo salar*	*Chordata Actinopterygii*	NCBI	XP_014048167.1	ATP13A1	
Salmo salar B1	*Salmo salar*	*Chordata Actinopterygii*	NCBI	XP_013990823.1	ATP13A2	
Salmo salar B2	*Salmo salar*	*Chordata Actinopterygii*	NCBI	XP_014045339.1	ATP13A3	
Salmo salar B4	*Salmo salar*	*Chordata Actinopterygii*	NCBI	XP_014004753.1	ATP13A3	
Salmo salar B3	*Salmo salar*	*Chordata Actinopterygii*	NCBI	XP_014071623.1	ATP13A3	
Cynoglossus semilaevis A1	*Cynoglossus semilaevis*	*Chordata Actinopterygii*	NCBI	XP_008327824.1	ATP13A1	
Cynoglossus semilaevis B1	*Cynoglossus semilaevis*	*Chordata Actinopterygii*	NCBI	XP_008316831.1	ATP13A2	
Cynoglossus semilaevis B3	*Cynoglossus semilaevis*	*Chordata Actinopterygii*	NCBI	XP_008328993.1	ATP13A3	
Cynoglossus semilaevis B2	*Cynoglossus semilaevis*	*Chordata Actinopterygii*	NCBI	XP_008332305.1	ATP13A3	
Saccoglossus kowalevskii B1	*Saccoglossus kowalevskii*	*Hemichordata Enteropneusta*	NCBI	XP_006819618.1	-	
Saccoglossus kowalevskii B2	*Saccoglossus kowalevskii*	*Hemichordata Enteropneusta*	NCBI	XP_006811882.1	-	
Callorhinchus milii B1	*Callorhinchus milii*	*Chordata Chondrichthyes*	NCBI	XP_007905146.1	ATP13A2	
Callorhinchus milii B2	*Callorhinchus milii*	*Chordata Chondrichthyes*	NCBI	XP_007900608.1	ATP13A3	
Latimeria chalumnae A1	*Latimeria chalumnae*	*Chordata Sarcopterygii*	NCBI	XP_005991859.1	ATP13A1	

Latimeria chalumnae B3	*Latimeria chalumnae*	*Chordata Sarcopterygii*	NCBI	XP_005989474.1#	-	# Gaps in sequence filled (see table text)
Latimeria chalumnae B2	*Latimeria chalumnae*	*Chordata Sarcopterygii*	Ensembl	ENSLACT00000014377	ATP13A3	N-terminal sequence 1-80 ending ATWAEE taken from XP_005999851.2
Latimeria chalumnae B1	*Latimeria chalumnae*	*Chordata Sarcopterygii*	NCBI	XP_014344004.1	ATP13A2	
Xenopus laevis A1	*Xenopus laevis*	*Chordata Amphibia*	KEGG	xla:100158446	ATP13A1	
Xenopus laevis B1	*Xenopus laevis*	*Chordata Amphibia*	KEGG	xla:446724	-	
SPF1	*Saccharomyces cerevisiae*	*Fungi Ascomycota*	Uniprot	P39986	ATP13A1	
YPK9	*Saccharomyces cerevisiae*	*Fungi Ascomycota*	Uniprot	Q12697	n.s.	
